# The gut microbiota and sleep in infants: a focus on diurnal rhythmicity patterns

**DOI:** 10.1080/29933935.2026.2649096

**Published:** 2026-03-29

**Authors:** Fannie Kerff, Christophe Mühlematter, Anja Adamov, Deborah Fast, Serafina Plüss, Petra Zimmermann, Salome Kurth, Nicholas A. Bokulich

**Affiliations:** a Department of Health Sciences and Technology, ETH Zurich, Zurich, Switzerland; b Department of Psychology, University of Fribourg, Fribourg, Switzerland; c Faculty of Health Science and Medicine, University of Lucerne, Lucerne, Switzerland; d Department of Paediatrics, Children's Hospital of Central Switzerland, Lucerne, Switzerland; e Department of Community Health, Faculty of Science and Medicine, University of Fribourg, Fribourg, Switzerland; f Department of Pulmonology, University Hospital Zurich, Zurich, Switzerland

**Keywords:** Gut microbiota, infant sleep, microbiota–gut–brain axis, melatonin, diurnal rhythmicity

## Abstract

Emerging evidence supports a bidirectional relationship between the gut microbiome and sleep, which is partly mediated by the microbiota‒gut‒brain axis. Infancy is a critical window for the establishment of both the gut microbiome and sleep regulation, which we hypothesize to be linked across both short (diurnal) and long (monthly) time scales. In this longitudinal study, we investigated associations between gut microbiota development and sleep patterns in 20 infants at 2, 4, and 6 months of age (*n* = 163 samples). Infants were continuously monitored across 48-h sampling periods. The gut microbiota profiles were characterized using 16S rRNA gene sequencing; gut melatonin concentrations were measured; sleep data were collected via wearable actimetry, 24-h parent-reported sleep diaries, and the Brief Infant Sleep Questionnaire; and parenting style and behavioral development were assessed. In some infants, bacterial diversity followed diurnal rhythmic patterns. While bacterial rhythmicity was not significantly associated with sleep rhythmicity, infants with higher microbial alpha diversity showed more robust sleep patterns. Infant age emerged as the strongest predictor of gut microbial diversity and melatonin levels. Our findings suggest that gut microbial establishment may support the maturation of sleep‒wake rhythms in early infancy. Further research is needed to elucidate mechanistic roles of the gut microbiome in sleep development.

## Introduction

Microbial colonization of the gastrointestinal tract begins at birth and is influenced by multiple factors including maternal microbiota, gestational age, mode of delivery, feeding practices (breastfeeding *versus* formula), and antibiotic exposure.^1-3^ During the first 1000 d of life, the gut microbiome undergoes its most rapid period of development, playing a critical role in immune, endocrine, and metabolic functions, and other host developmental processes.^4^

Growing evidence suggests that the gut microbiome is associated with both sleep^5^ and cognitive abilities.^6^ Notably, the first year of life—a period of development and stabilization of the gut microbiome—coincides with important milestones in sleep regulation and neurodevelopment.^7^^,^^8^ In particular, brain oscillations during sleep actively regulate neuroplasticity, thereby supporting learning and healthy neurodevelopment.^9^ Previous studies have linked behavioral and neurophysiological patterns of sleep with neuronal connectivity.^10^^,^^11^ The bidirectional microbiome–sleep relationship is partially mediated by the microbiota–gut–brain axis and is shaped by environmental and lifestyle factors.^5^

The development of sleep regulation primarily includes a gradual establishment of rhythm, including for example increased consolidation of sleep and wake bouts, increasing preference towards nighttime sleep and less fragmented nighttime sleep (all along a continuum). Several pathways of gut microbiota‒host interaction suggest a possible influence on sleep development. For instance, in infants, human milk oligosaccharides (HMOs) promote the growth of *Bifidobacterium*^12^, which produce short-chain fatty acids (SCFAs), such as propionate.^13^ Fecal propionate levels have been positively associated with sleep duration in infants,^14^ supporting the relationship between microbial metabolites and early-life sleep regulation. Similarly, infants receiving a prebiotic blend of indigestible oligosaccharides in infant formula have shown faster consolidation of daytime waking periods^15^ and potential beneficial effects on sleep duration,^16^ suggesting beneficial roles of *Bifidobacterium* and SCFAs production, although further clarification on probiotics is needed.^17^^,^^18^ Recent findings also indicate that infants' sleep–wake patterns are associated with regularity of feeding schedules.^19^ Since certain gut bacterial taxa show diurnal oscillations,^20^ and several SCFAs fluctuate rhythmically throughout the day,^21^ it is possible that the systemic circadian rhythm plays a role in the molecular mechanisms underlying sleep–microbiome interactions.

While systemic melatonin is recognized for its crucial role in regulating circadian rhythm, research on intestinal melatonin and associations with gut bacteria is only emerging.^22^^,^^23^ Additionally, small amounts of melatonin are present in certain foods (e.g., in pistachios, cherries, and grapes), which may also contribute to intestinal and systemic melatonin levels.^24^ Even though the pineal gland, producing melatonin, and melatonin receptors are present at birth, the ability of the gland to release and produce melatonin rhythmically is just developing during the first year of life.^25^^,^^26^ Using a cross-sectional approach, it was proposed that fecal melatonin increases with age (high inter-individual variability), with higher levels earlier in the day (diurnal variations), and higher levels with more time passed since the last bowel movement (accumulation of melatonin).^22^ Still, contrasting evidence on diurnal fluctuations has been reported, and the specific pathways and bacterial species involved in melatonin metabolism (both production and degradation) in the gut remain unclear.^23^

Despite evidence that gut microbiota diversity is associated with sleep in adults^27-30^ and school-aged children,^31-36^ very few studies have examined microbiota-sleep interactions in infancy (0–1 y). Among these, longitudinal studies in infants have shown links between gut bacterial diversity and the maturation of behavioral sleep patterns, including nighttime preference for sleep, but also neurophysiological markers of sleep homeostasis (slow wave activity).^10^ Moreover, emerging evidence suggests that diurnal rhythmicity patterns already develop in the gut microbiota and are impacted by the diet^37^ and intertwined with the host's circadian rhythm.^20^ Specifically, diurnal oscillations have been detected across dominant genera, including *Bifidobacterium*, *Bacteroides*, *Veillonella*, *Streptococcus*, and *Clostridium*, with the number of rhythmic bacteria gradually increasing as the infant gut matures and a regular diet is introduced.^37^

Altogether, despite infancy being a critical period for both microbiome development and sleep regulation establishment, the role of gut bacteria in modulating infant sleep remains underexplored. Notably, high-frequency sampling is needed to capture time series and potential rhythmicity across the 24-h day, and only longitudinal trajectories adequately capture the rapid and multi-dimensional transitions in the infant gut microbiota and sleep. To address this gap, our study focused on gut microbiota diurnal rhythmicity patterns, leveraging repeated sampling over 48-h periods per infant to provide novel insights into these temporal dynamics. Specifically, this study aimed to explore (1) microbial rhythmicity, (2) temporal volatility patterns (bacterial community variability over time) along age, (3) quantify the effect of feeding and sleep history on the microbiota, and (4) determine the link between gut microbiota patterns and sleep maturation. While the rhythmicity and volatility analyses are exploratory, we hypothesize that the gut microbiome and sleep rhythm establishment are linked on a short-term (diurnal) time scale, with gut bacterial rhythmicity strengthening with age, and that infants with a more volatile gut bacterial profile exhibit a delayed establishment of sleep maturation. Building upon previous studies in older children, we also aimed to replicate and extend findings regarding overall diversity; we hypothesize that infants with a less diverse microbiota would exhibit delayed sleep maturation across the first six months of life, as it has already been captured later in life. With the aim of advancing microbiome-based therapies to improve sleep, this study focuses on how the gut microbiome influences sleep, rather than vice versa.

## Methods

### Study design

We launched an observational longitudinal cohort study to investigate the synchronization of the gut microbiota and sleep in infancy. From May 2023 to April 2024, this study followed 20 healthy term-born infants across development, with assessments scheduled at 2, 4, and 6 months of age. The inclusion criteria required infants to be generally healthy, vaginally delivered, and primarily breast-fed at the time of enrollment.

All procedures were conducted in accordance with the Declaration of Helsinki and approved by the responsible cantonal ethics committee (BASEC 2019-02250). Written parental consent was obtained after explanation of the study procedures and before effective enrollment. Families received non-monetary gifts to maintain adherence across assessments.

### Experimental design

**Figure 1.**
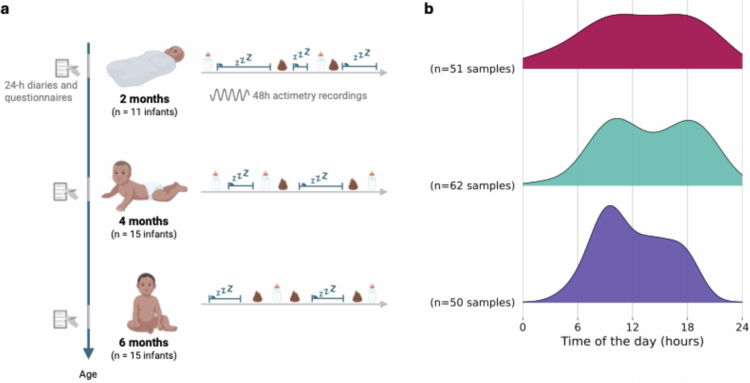
Study overview. (a) Study design. (b) Stool sample collection density according to clock times across age assessments (i.e., ages 2 months, 4 months, and 6 months).

The gut microbiota was evaluated through biological sampling (stool); infant sleep was assessed with objective (actimetry via wearable devices) and subjective (24-h diaries and questionnaires) parameters. A graphic study design is displayed in Figure 1a.

#### Stool sample collection

Parents collected stool samples from the infant's diaper at all ages using sterile disposable pipettes or laboratory spatulas.^10^ Samples were kept in 5 mL Eppendorf tubes, placed in Whirlpak bags and temporarily stored in the families' refrigerator, until transported to the laboratory within 76 h in cooling boxes for temperature maintenance. Aliquots were generated and stored at −80 °C.

#### Sleep data collection

At each age assessment, continuous sleep data were collected across several days using ankle actimetry (GENEactiv accelerometer, Activinsights Ltd., Kimbolton, UK 43 × 40 × 13 mm, micro-electro-mechanical systems sensor, 16 g, 30 Hz frequency) combined with a 24-h sleep–wake diary, which was adapted from Werner et al.^38^. Parents documented actimeter removal in the diary, which included detailed 15-min interval reporting of sleep or wake periods, feeding times, crying, and bedtimes. Using data from both actimetry and the 24-h diary, sleep and wake periods and feeding times were identified following our laboratory's established standards.^19^^,^^39^ Additionally, the Brief Infant Sleep Questionnaire (BISQ), a clinically-validated parent-reported questionnaire capturing infants/toddler (0–29 months) habitual sleep over a full week, was used at all ages.^40^

#### Additional questionnaires and feeding diary

Demographic information was provided by parents in an online survey at each assessment, which was generated using SoSci Survey. Further, at all ages, parents filled a parenting style questionnaire (Baby Care Questionnaire, BCQ),^41^ as well as standardized questionnaires on behavioral development (Ages and Stages Questionnaire, ASQ).^42^ Feeding times (clock times of infant meals) were reported by the parents in the sleep diary at all ages.

### Data processing

#### Infant gut microbiota

##### Stool processing: DNA extraction and 16S rRNA gene sequencing.

DNA was extracted from approximately 80 mg (60 to 100 mg accepted; 80 µL if sample was liquid) of stool using MagMAX™ Microbiome Ultra Nucleic Acid Isolation Kit with bead plates following the manufacturer's instructions with one modification: bead beating 2 × 5 min at 30 Hz. 400 µL of lysate were transferred to the KingFisher Deep Well Plates and stored at −20 °C. DNA extraction was done on the KingFisher Apex (binding, washing, elution). Eluted DNA was collected in KingFisher standard plates and stored at 4 °C (refrigerator) overnight.

Amplicon sequencing was performed on the Illumina NextSeq 2000 platform. We used the HighALPS ultra-high throughput library preparation protocol^43^ to profile bacterial communities via sequencing the V4 region of the 16S rRNA gene using 515F and 806R primers.^44^^,^^45^ DNA from each sample was amplified using barcoded forward and reverse primers targeting the 16S rRNA gene (1-step PCR; 34 cycles). The PCR products were cleaned with 0.7× AMPure XP beads on the KingFisher Apex, quantified with the Qubit assay, combined into one final pool after serial dilutions, and manually cleaned with 0.7× beads, followed by an elution in 100 µL nuclease-free water. Qubit (dsDNA HS Assay) and TapeStation (HS D1000) analyses confirmed sufficient concentration and quality of the final pool, which was submitted for library pooling.

##### Sequences processing: gut microbiota composition and diversity.

Sequence data were analyzed using QIIME 2^46^ Sequences were demultiplexed, denoised (truncation at 150 bp using the q2-dada2 plugin^47^) and rarefied at a sequencing depth of 3035 reads. Features with a frequency less than 10 across all samples were discarded. Taxonomic classification was performed using a naive Bayes classifier^48^ trained on the V4 region extracted from SILVA version 138.1^49^^,^^50^ (using RESCRIPt^51^), and sequences not assigned to a phylum were removed. Finally, diversity metrics were computed using the q2-boots plugin,^52^ averaging the metrics over 100 computations. Calculated alpha diversity metrics included observed features, Shannon entropy, Pielou evenness and Faith phylogenetic diversity; the beta diversity metrics measured were Bray‒Curtis dissimilarity,^53^ Jaccard similarity index,^54^ unweighted UniFrac^55^ and weighted UniFrac.^56^

##### Gut microbiota rhythmicity.

To detect 24-h oscillations, bacterial rhythmicity per infant and age was assessed through cosine fittings of bacterial alpha diversity and individual bacterial genera (relative abundance), meeting the minimum requirement of four samples per age, following the equation:

(1) $$\begin{array}{l} f\left(x\right)=A\times \cos\left(\frac{2\pi }{\lambda }x+\;\phi \right)+C,\\ \mathrm{with}\;A=\mathrm{amplitude},\;\lambda =\mathrm{period}=24\;h,\;\phi =\mathrm{phase}\;\mathrm{shift},\;\\ C=\mathrm{constant},\;\mathrm{and}\;x=\mathrm{samples},\mathrm{with}\geq 4\;\mathrm{samples}\;\mathrm{required}. \end{array}$$

Bacterial diurnal rhythmicity was quantified with *R*^2^ (*R*-squared) values computed for each cosine fit. High *R*^2^ values represented a good cosine fit over time (i.e., high diurnal rhythmicity).

##### Gut microbiota temporal volatility.

For each infant, we quantified the variability of the gut bacterial community over time (48 h) at each age. To do this, the centroid of each infant was defined for each beta diversity metric at each age. The centroid was computed as the mean position of the three first principal components of those samples. The gut bacterial variability of each infant at each age was estimated by measuring the median distance of the samples to their corresponding centroid (i.e., the centroid of the (≥2) samples of that infant at that age). This measure of variability in the microbiota of an infant (across 48 h) is henceforth referred to as the gut bacterial “temporal volatility” of the infant at that age. A high volatility indicates that an individual's gut microbiota experiences rapid change over the measurement period (in this case, the 48-h sampling period), whereas a low volatility indicates that the community was highly stable over that period.

#### Gut melatonin

Samples with enough remaining stool were analyzed for melatonin. The gut melatonin concentration was determined using a radioimmunoassay (RIA) RK-MEL2, manufactured by NovoLytiX GmbH (Witterswil, Switzerland) following the manufacturer's instructions (IFU, version 2022-03-07) with specific adaptations for the stool samples. For each sample, 50 mg of stool was homogenized in 1 mL of RK-MEL2 incubation buffer; the mixture was vigorously vortexed for 10 s and left for 10 min at room temperature to maximize homogenization. The latter step was repeated 3×, and the homogenate was centrifuged at 13,000 rpm (7000 × *g*) for 10 min in an Eppendorf Minifuge; the same centrifugation procedure was done to the supernatant. Finally, the supernatant was used for RIA, with a correction factor of 0.77 (as established by standards^57^) to adjust for matrix effects, which was validated through spiking recovery experiments. The measured gut melatonin levels reflect the net balance of its metabolism—including both production and degradation—within the infant gut environment.

#### Infant sleep

To characterize infant sleep patterns, we used two distinct proxies: the Circadian Function Index (CFI), derived from objective actimetry data, and the infant sleep quality index (BabySQUID), a composite score based on the sleep questionnaire (BISQ).

##### Sleep rhythmicity: circadian function index (CFI).

Based on actimetry data, the CFI was computed, which measures the regularity of activity-rest patterns with a score from 0 to 1,^58^ and was used as a proxy for sleep‒wake rhythm maturation for each infant at each age.^20^ The CFI was quantified for infants who were assessed during at least three continuous days at an age. The CFI is defined as:

(2) $$\begin{array}{l} \mathrm{CFI}=\;\frac{\mathrm{IS}+\frac{2\;-\mathrm{IV}}{2}+\mathrm{RA}}{3},\;\\ \mathrm{with}\;\mathrm{IS}=\mathrm{interdaily}\;\mathrm{stability},\;\mathrm{IV}=\mathrm{intradaily}\;\mathrm{variability}\;\mathrm{and}\;\mathrm{RA}=\mathrm{relative}\;\mathrm{amplitude}. \end{array}$$

Interdaily stability (IS) measures how consistent activity patterns are across days; a higher IS indicates more regular, stable daily rhythms. The intradaily variability (IV) indicates how often transitions occur between rest and activity within a day; a higher IV means more fragmented patterns; lower IV reflects more consolidated periods. The relative amplitude (RA) reflects the difference between peak activity and rest; higher RA shows a clearer distinction between active and rest periods. The CFI is henceforth referred to as “sleep rhythmicity”.

##### Sleep quality: infant sleep quality index (BabySQUID).

The BabySQUID was determined for each age as the composite score of four variables derived from the BISQ sleep questionnaire: nighttime sleep duration, sleep onset latency, bedtime and number of nighttime awakenings. The BabySQUID estimated sleep quality over one week. Infants with maximally two missing infant sleep variable(s) were still included, with the missing values replaced by the median of that variable. All variables were min-max scaled, then all variables except nighttime sleep duration were reverse-coded (sleep onset latency, bedtime and number of nighttime awakenings), and the normalized values were averaged with equal weights to produce a single score from 0 to 1, reflecting overall sleep behavior. In this context, we hypothesized that earlier bedtimes and shorter sleep onset latencies generally serve as indicators of more consolidated sleep patterns. This is supported by evidence linking earlier bedtimes to stronger neuronal connectivity in infants,^11^ and the assumption that, within this developmental window, shorter latency typically reflects higher sleep efficiency rather than extreme sleep deprivation.

#### Other variables

We used parent-reported diaries to determine the infant's sleep and feeding history prior to each stool sample. To estimate the sleep history, we compared the stool sample clock time with the parent-rated entries (nighttime sleep and awakenings, wake-up time, and daytime naps) to assess the time spent awake prior to each sampling. Then, we used the related diary information on falling asleep times and naps to estimate the duration of that last sleep. The same methodology was used to estimate the feeding history (time since the last feeding) using the parent-rated feeding clock times in the diaries. Finally, feeding rhythmicity was estimated by calculating the standard deviation of the intervals between feedings for each infant for each day at each age assessment.

The parenting style (attuned *versus* structured) was estimated using the attunement score of the BCQ questionnaire. Attunement represents reliance on infant cues and close physical contact; parents with a more attuned caring/parenting style would be more prone to change their behavior based on the needs of the infant. The infant behavioral developmental stage was estimated using the composite score (sum) of the five sub-scores of the ASQ questionnaire.

### Statistical analysis

In this study, we investigated the relationship between the gut microbiota and infant sleep using several complementary models. First, we assessed novel microbial temporal patterns at an age-level resolution—specifically microbiota rhythmicity and temporal volatility—providing novel insights, while also replicating previous findings on the associations between alpha diversity and sleep. Second, we performed sample-level resolution analyses to investigate immediate physiological associations, including gut melatonin levels, and the acute effects of sleep and feeding history on microbiota alpha diversity. This dual-resolution approach allowed us to capture both the broad developmental trajectories of the infant and the high-resolution associations between specific microbial and hormonal biomarkers.

We used linear mixed models (LMMs) to account for repeated measures, irregular sampling, and potential confounding factors in the longitudinal microbiota data. For all analyses, missing values in exposure or outcome variables led to the exclusion of that sample/age for that analysis, while missing variables in the covariates were replaced by the median value of that variable. Note that each infant's gut microbiota diversity at a given age was estimated using the median of their samples' alpha diversity values at that age. Statistical significance was determined at the 5% level.

#### Diurnal patterns along age

To investigate the links between bacterial diurnal rhythmicity patterns in the gut microbiota and infant sleep rhythmicity, LMMs were created adjusting for the infants' repeated measures, feeding rhythmicity, parenting style, age and sex, following the equation:

(3) $$\begin{array}{l} \mathrm{sleep}\;{\mathrm{rhythmicity}}_{\mathrm{CFI}}=f\left(\mathrm{gut}\;\mathrm{bacteria}\;\mathrm{rhythmicity}\right)\\ \sim \;\mathrm{gutbacteria}\;{\mathrm{rhythmicity}}_{\mathrm{metric}}+\mathrm{feeding}\;{\mathrm{rhythmicity}}_{\mathrm{std}\;\mathrm{of}\;\mathrm{hours}\;\mathrm{between}\;\mathrm{feedings}}+\mathrm{parenting}\;{\mathrm{style}}_{\mathrm{BCQ}\;\mathrm{score}}+{\mathrm{age}}_{\mathrm{days}}+\mathrm{sex},\;\\ \begin{array}{l} \mathrm{with}\;\mathrm{metric}=R^{2}\;\mathrm{of}\;\mathrm{cosinefit}\;\mathrm{of}\;\mathrm{diversity}\;\mathrm{metrics}(\mathrm{observed}\;\mathrm{features},\\ \mathrm{Shannon}\;\mathrm{entropy},\mathrm{Pielou}\;\mathrm{evenness}\;\mathrm{and}\;\mathrm{Faith}\;\mathrm{phylogenetic}\;\mathrm{diversity})\;\\ \mathrm{and}\;\mathrm{individual}\;\mathrm{genera}\;(\mathrm{relative}\;\mathrm{abundance}\;\mathrm{of}\;\mathrm{most}\;\mathrm{abundant}\;\mathrm{genera}). \end{array} \end{array}\;$$

This modeling approach allows us to determine whether sleep maturation—as reflected in sleep rhythmicity—is associated with bacterial rhythmicity, independent of the influence of external factors like feeding intervals or parental care.

Gut microbiota temporal volatility was then investigated at each age, controlling for the infants' repeated measures, age and sex.

#### Modulation of infant sleep by the gut microbiota

To investigate the modulation of infant sleep by the gut microbiota, we examined the associations between gut microbial diversity and temporal volatility and the maturation of infant sleep patterns, including rhythmicity and quality, while adjusting for environmental and developmental confounders such as parenting style and behavioral stage.

##### Sleep rhythmicity: CFI.

Two LMMs were created adjusting for the infants' repeated measures, as well as parenting style, infant behavioral developmental stage, age, and sex, following the equations:

(4) $$\begin{array}{l} \mathrm{sleep}\;{\mathrm{rhythmicity}}_{\mathrm{CFI}}\;=f\left(\mathrm{gut}\;\mathrm{bacteria}\;\mathrm{diversity}\right)\\ \sim \;\mathrm{gut}\;\mathrm{bacteria}\;{\mathrm{diversity}}_{\mathrm{metric}}+\;\mathrm{parenting}\;{\mathrm{style}}_{\mathrm{BCQscore}}\\ \begin{array}{l} +\;\mathrm{infant}\;{\mathrm{development}}_{\mathrm{ASQscore}}+{\mathrm{age}}_{\mathrm{days}}+\mathrm{sex},\\ \mathrm{with}\;\mathrm{metric}=\mathrm{median}\;\mathrm{of}\;\mathrm{diversity}\;\mathrm{metrics}(\mathrm{observed}\;\mathrm{features},\\ \mathrm{Shannon}\;\mathrm{entropy},\mathrm{Pielou}\;\mathrm{evenness}\;\mathrm{and}\;\mathrm{Faith}\;\mathrm{phylogenetic}\;\mathrm{diversity}). \end{array} \end{array}\;$$

(5) $$\begin{array}{l} \mathrm{sleep}\;{\mathrm{rhythmicity}}_{\mathrm{CFI}}\;=f\left(\mathrm{gut}\;\mathrm{bacteria}\;\mathrm{temporal}\;\mathrm{volatility}\right)\\ \sim \;\mathrm{gut}\;\mathrm{bacteria}\;\mathrm{temporal}\;{\mathrm{volatility}}_{\mathrm{metric}}+\;\mathrm{parenting}\;{\mathrm{style}}_{\mathrm{BCQscore}}\\ \begin{array}{l} +\;\mathrm{infant}\;{\mathrm{development}}_{\mathrm{ASQscore}}+{\mathrm{age}}_{\mathrm{days}}+\mathrm{sex},\\ \mathrm{with}\;\mathrm{metric}=\mathrm{median}\;\mathrm{distance}\;\mathrm{to}\;\mathrm{centroid}\;\mathrm{using}\;\mathrm{Bray}-\mathrm{Curtis}\;\mathrm{dissimilarity},\;\\ \mathrm{Jaccard}\;\mathrm{similarity}\;\mathrm{index},\mathrm{unweighted}\;\mathrm{UniFrac}\;\mathrm{and}\;\mathrm{weighted}\;\mathrm{UniFrac}\;\mathrm{distances}. \end{array} \end{array}$$

##### Sleep quality: BabySQUID.

Two LMMs were then created adjusting for the infants' repeated measures, as well as parenting style, infant behavioral developmental stage, age, and sex, following the equations:

(6) $$\begin{array}{l} \mathrm{sleep}\;{\mathrm{quality}}_{\mathrm{babySQUID}}\;=f\left(\mathrm{gut}\;\mathrm{bacteria}\;\mathrm{diversity}\right)\\ \sim \mathrm{gut}\;\mathrm{bacteria}\;{\mathrm{diversity}}_{\mathrm{metric}}+\;\mathrm{parenting}\;{\mathrm{style}}_{\mathrm{BCQ}\;\mathrm{score}}\\ \begin{array}{l} +\;\mathrm{infant}\;{\mathrm{development}}_{\mathrm{ASQ}\;\mathrm{score}}+{\mathrm{age}}_{\mathrm{days}}+\mathrm{sex},\\ \mathrm{with}\;\mathrm{metric}=\mathrm{median}\;\mathrm{of}\;\mathrm{diversity}\;\mathrm{metrics}(\mathrm{observed}\;\mathrm{features},\;\\ \mathrm{Shannon}\;\mathrm{entropy},\mathrm{Pielou}\;\mathrm{evenness}\;\mathrm{and}\;\mathrm{Faith}\;\mathrm{phylogenetic}\;\mathrm{diversity}). \end{array} \end{array}$$

(7) $$\begin{array}{l} \mathrm{sleep}\;{\mathrm{quality}}_{\mathrm{babySQUID}}\;=f\left(\mathrm{gut}\;\mathrm{bacteria}\;\mathrm{temporal}\;\mathrm{volatility}\right)\\ \sim \;\mathrm{gut}\;\mathrm{bacteria}\;\mathrm{temporal}\;{\mathrm{volatility}}_{\mathrm{metric}}+\;\mathrm{parenting}\;{\mathrm{style}}_{\mathrm{BCQ}\;\mathrm{score}}\\ \begin{array}{l} +\;\mathrm{infant}\;{\mathrm{development}}_{\mathrm{ASQ}\;\mathrm{score}}+{\mathrm{age}}_{\mathrm{days}}+\mathrm{sex},\\ \mathrm{with}\;\mathrm{metric}=\mathrm{median}\;\mathrm{distance}\;\mathrm{to}\;\mathrm{centroid}\;\mathrm{using}\;\mathrm{Bray}-\mathrm{Curtis}\;\mathrm{dissimilarity},\;\\ \mathrm{Jaccard}\;\mathrm{similarity}\;\mathrm{index},\mathrm{unweighted}\;\mathrm{UniFrac}\;\mathrm{and}\;\mathrm{weighted}\;\mathrm{UniFrac}\;\mathrm{distances}. \end{array} \end{array}$$

To explore the effect of microbial composition on sleep quality, random forest classifiers were trained to predict the BabySQUID score binarized at the median. This approach complements the LMMs, which assume linear relationships, by capturing non-linear complex (high-dimensional) interactions between the gut microbiota and sleep patterns. Input features included (1) the ASV relative abundance table, or (2) its k-merization,^59^ selecting the relative abundance of the top features based on their frequency or TF-IDF score. K-mers length (i.e., *n*-gram size) was set to 16, as done in previous work.^59^ Models were evaluated using repeated 80/20 train-test splits via different cross-validation methods from Scikit-learn.^60^ Accuracy and weighted F1 (harmonic mean of precision and recall) scores were averaged across the splits.

#### Exploratory analysis of the samples

##### Gut melatonin.

As an exploratory analysis, we tested the associations between the abundance of gut melatonin in the samples (in pg/g of stool)—the primary sleep-regulating hormone in the human body—and the respective time since the last bowel movement (i.e., the time since the last stool sample), as well as the immediate prior time spent awake (sleep history) and the time since the infant was last fed. A LMM was performed on the samples, adjusting for infants' repeated measures, age and sex:

(8) $$\begin{array}{cl} \mathrm{gut}\;{\mathrm{melatonin}}_{\frac{\mathrm{pg}}{g\;\mathrm{of}\;\mathrm{stool}}} & \;\sim \;\mathrm{time}\;\mathrm{since}\;\mathrm{last}\;\mathrm{bowel}\;{\mathrm{movement}}_{h}+\mathrm{prior}\;\mathrm{time}\;\mathrm{spent}\;{\mathrm{awake}}_{h}\\ & +\;\mathrm{time}\;\mathrm{since}\;\mathrm{last}\;\mathrm{feedin}g_{h}+\mathrm{ag}e_{\mathrm{days}}+\mathrm{sex}. \end{array}$$

To identify specific microbial drivers associated with gut melatonin, differential abundance analyses between all microbiota genera and families and gut melatonin levels were performed using ANCOM-BC2,^61^ following a 10% prevalence filter (within the QIIME 2 framework). The model adjusted for infants' repeated measures, age, and sex. Statistical significance was defined as a *p*-value < 0.05 and a *q*-value < 0.1, after applying a Benjamini‒Hochberg adjustment for multiple testing.^62^

##### Effects of sleep and feeding history on the gut microbiota

To identify which directly preceding sleep and feeding history factors are predominantly associated with the infant gut microbiota, LMMs for diversity metrics were created, adjusting for the infants' repeated measures, as well as age and sex, following the equation:

(9) $$\begin{array}{l} \mathrm{gut}\;\mathrm{microbial}\;{\mathrm{diversity}}_{\mathrm{metric}}\;\sim \;\mathrm{prior}\;\mathrm{time}\;\mathrm{spent}\;{\mathrm{awake}}_{h}+\mathrm{duration}\;\mathrm{of}\;\mathrm{last}\;{\mathrm{sleep}}_{h}\\ +\mathrm{time}\;\mathrm{since}\;\mathrm{last}\;{\mathrm{feeding}}_{h}+{\mathrm{age}}_{\mathrm{days}}+\mathrm{sex},\\ \begin{array}{l} \mathrm{with}\;\mathrm{metric}=\mathrm{observed}\;\mathrm{features},\mathrm{Shannon}\;\mathrm{entropy},\\ \mathrm{Pielou}\;\mathrm{evenness}\;\mathrm{and}\;\mathrm{Faith}\;\mathrm{phylogenetic}\;\mathrm{diversity}. \end{array} \end{array}$$

## Results

### Study cohort and microbiota composition

In total, 163 stool samples from 20 infants were included (two samples had missing collection dates and times, and 24 samples had a sequencing depth below 3035 reads, leading to their exclusion; Supplementary Figure 1). Most infants (*n* = 11) were monitored at two ages, while five infants were assessed at all ages, and four infants only had data at one age (2 months: *n* = 11; 4 months: *n* = 15; 6 months: *n* = 15) (Figure 1, Table 1, Supplementary Figure 2). The timing of stool samples narrowed with age; while samples from younger infants were spread throughout the 24-h cycle, samples from older infants (6 months) were more concentrated during daylight hours, with a pronounced morning peak where 50% of samples were collected between 9:15 AM and 2:54 PM (Table 1, Figure 1b). The gut microbiota of the infant cohort included 426 features from 140 genera, from 10 phyla with *Firmicutes* and *Proteobacteria* being the dominant phyla (Supplementary Figures 3 and 4), with some inter-individual variability (Supplementary Figure 5). The detailed distributions of the scores derived from the parent-rated questionnaires on infant sleep (BISQ), parenting style (BCQ), and behavioral development (ASQ) are included in Table 1 and Supplementary Figures 6–8. Most infants remained healthy and antibiotic-free throughout the study period. Minor viral infections were reported for two participants, neither of whom required antibiotic treatment. One participant (infant 17) received antibiotics (amoxicillin and azithromycin) at 6 months of age to treat otitis. As sensitivity analyses showed no impact on the primary microbiome‒sleep associations, data from infant 17 at 6 months was retained to maximize sample size.

**Table 1.** Infants' samples characteristics. Characteristics of the 163 included samples. Unless otherwise indicated, values represent median (interquartile range). *Escherichia-S* refers to *Escherichia-Shigella*; *Clostridium* refers to *Clostridium sensu stricto 1*.

	2 months (*n*_samples_ = 51)	4 months (*n*_samples_ = 62)	6 months (*n*_samples_ = 50)
Infants, *n*	11	15	15
Twins, *n*	2	2	0
Female, *n*_samples_ (%)	38 (74.5)	25 (40.3)	19 (38.0)
Age in days	76.0 (71.0, 81.0)	128 (126, 143)	190 (185, 200)
Time of the day (h)	13.7 (8.80, 17.8)	13.7 (10.0, 18.2)	11.2 (9.25, 14.9)

**Gut microbiota**			
Alpha diversity			
Observed features	22.0 (19.0, 29.5)	26.5 (21.0, 30.0)	34.0 (26.0, 39.1)
Shannon entropy	2.40 (1.92, 2.81)	2.40 (2.08, 2.90)	2.78 (2.24, 3.22)
Pielou evenness	0.532 (0.447, 0.588)	0.528 (0.457, 0.594)	0.546 (0.469, 0.622)
Faith phylogenetic diversity	2.31 (2.13, 2.96)	2.55 (2.24, 3.11)	3.08 (2.37, 3.55)
Microbial rhythmicity	(*n* = 7)	(*n* = 6)	(*n* = 8)
Cosine fit of observed features	0.396 (0.113, 0.573)	0.152 (0.0535, 0.236)	0.486 (0.226, 0.834)
Cosine fit of Shannon entropy	0.286 (0.138, 0.578)	0.316 (0.188, 0.366)	0.212 (0.131, 0.641)
Cosine fit of Pielou evenness	0.304 (0.228, 0.540)	0.433 (0.268, 0.577)	0.304 (0.0920, 0.646)
Cosine fit of Faith phylogenetic diversity	0.464 (0.102, 0.631)	0.111 (0.0452, 0.261)	0.393 (0.285, 0.597)
Cosine fit of *Bifidobacterium* rel. abund	0.335 (0.195, 0.555)	0.226 (0.136, 0.353)	0.0947 (0.0524, 0.313)
Cosine fit of *Veillonella* rel. abund.	0.246 (0.180, 0.472)	0.561 (0.316, 0.680)	0.236 (0.0830, 0.933)
Cosine fit of *Escherichia-S.* rel. abund.	0.441 (0.169, 0.577)	0.400 (0.215, 0.509)	0.514 (0.364, 0.800)
Cosine fit of *Bacteroides* rel. abund.	0.202 (0.0959, 0.610)	0.263 (0.0456, 0.439)	0.678 (0.260, 0.842)
Cosine fit of *Clostridium* rel. abund.	0.224 (0.191, 0.510)	0.305 (0.196, 0.684)	0.775 (0.140, 0.816)
Microbial temporal volatility	(*n* = 11)	(*n* = 13)	(*n* = 13)
Based on Bray–Curtis dissimilarity	0.0406 (0.0316, 0.0844)	0.0547 (0.0413, 0.121)	0.0505 (0.0239 0.0886)
Based on Jaccard similarity index	0.0644 (0.0409, 0.0712)	0.0598 (0.0527, 0.0698)	0.0461 (0.0375, 0.0598)
Based on Unweighted UniFrac distance	0.0639 (0.0556, 0.109)	0.0898 (0.0784, 0.103)	0.0556 (0.0389, 0.0976)
Based on Weighted UniFrac distance	0.0517 (0.0305, 0.0769)	0.0684 (0.0388, 0.0785)	0.0724 (0.0606, 0.0834)
			
**Sleep**			
Sleep quality	(*n* = 10)	(*n* = 11)	(*n* = 12)
Sleep quality index (BabySQUID)	0.521 (0.449, 0.563)	0.593 (0.530, 0.674)	0.606 (0.559, 0.729)
BISQ: nighttime sleep duration (h)	10.0 (9.00, 10.0)	10.5 (9.75, 11.3)	10.3 (10.0, 11.0)
BISQ: sleep onset latency (h)	0.333 (0.333, 0.688)	0.333 (0.250, 0.500)	0.333 (0.229, 0.500)
BISQ: bedtime (h)	21.0 (19.6, 21.2)	21.0 (20.3, 21.3)	20.1 (19.7, 20.6)
BISQ: number of nighttime awakenings	2.00 (2.00, 2.00)	2.00 (2.00, 2.00)	2.00 (2.00, 2.00)
Sleep rhythmicity	(*n* = 7)	(*n* = 9)	(*n* = 9)
Circadian function index (CFI)	0.580 (0.561, 0.635)	0.673 (0.655, 0.737)	0.610 (0.583, 0.692)
			
			
**Parenting style and infant development**	(*n* = 10)	(*n* = 11)	(*n* = 12)
BCQ: attuned care score	3.54 (3.31, 3.77)	3.46 (3.19, 3.62)	3.31 (3.23, 3.77)
ASQ: development composite score	283 (273, 289)	270 (265, 293)	263 (243, 270)
			
**Sleep and feeding history**			
	(*n*_samples_ = 38)	(*n*_samples_ = 53)	(*n*_samples_ = 36)
Sleep history (h)	0.517 (0.250, 1.00)	1.00 (0.500, 2.25)	1.25 (0.583, 1.83)
	(*n*_samples_ = 37)	(*n*_samples_ = 49)	(*n*_samples_ = 32)
Duration of last sleep (h)	1.00 (0.500, 1.75)	1.00 (0.750, 4.25)	0.750 (0.500, 9.56)
	(*n*_samples_ = 39)	(*n*_samples_ = 48)	(*n*_samples_ = 31)
Feeding history (h)	1.00 (0.250, 2.04)	0.708 (0.238, 1.77)	0.750 (0.208, 1.58)
			
			
**Melatonin**	(*n*_samples_ = 31)	(*n*_samples_ = 50)	(*n*_samples_ = 44)
Gut melatonin (pg/g of stool)	78.4 (67.4, 97.6)	83.8 (62.2, 128)	115 (69.1, 264)

**Figure 2.**
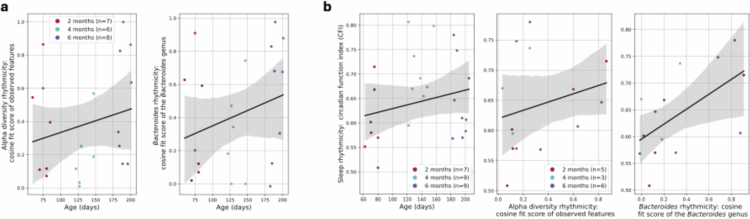
Gut microbiota rhythmicity *versus* sleep rhythmicity in infants. Regression lines (95% confidence intervals). (a) Rhythmicity in alpha diversity (observed features) (*p* > 0.05) and in the *Bacteroidetes* genus (*p* < 0.1) along age. (b) Sleep rhythmicity (CFI) along age, alpha diversity (observed features) rhythmicity and *Bacteroidetes* rhythmicity (all *p* > 0.05).

**Figure 3.**
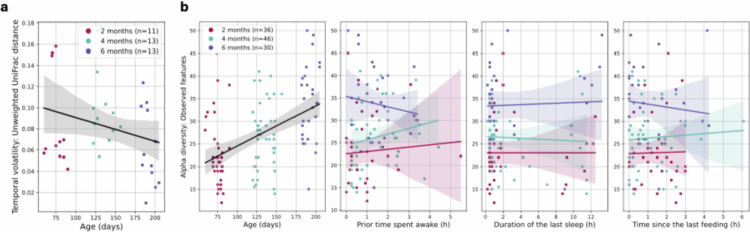
Gut microbiota temporal volatility and alpha diversity in infants. Regression lines (95% confidence intervals). (a) Microbial temporal volatility based on the unweighted UniFrac distance along age (*p* < 0.1). (b) Alpha diversity (observed features) along age (*p* < 0.001) and directly preceding sleep and feeding history (*p* > 0.05).

**Figure 4.**
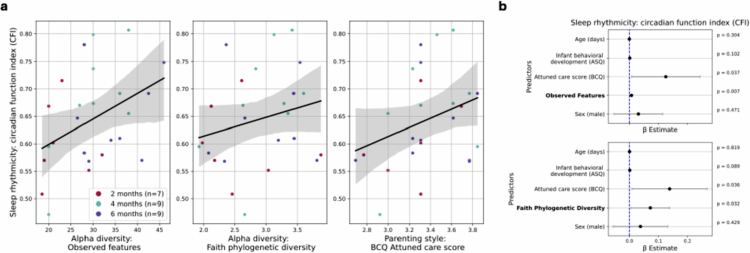
Sleep rhythmicity (CFI) in infants. Alpha diversity measured by observed features (feature richness) and Faith phylogenetic diversity (features biodiversity). Parenting style measured by the attunement score of the BCQ. (a) Regression lines (95% confidence intervals) of sleep rhythmicity along alpha diversity metrics. (b) Coefficient plots. Significant associations between observed features and Faith phylogenetic diversity, and the parenting style, with sleep rhythmicity (*p* < 0.05). Dots are the coefficients, and error bars represent the 95% confidence intervals.

**Figure 5.**
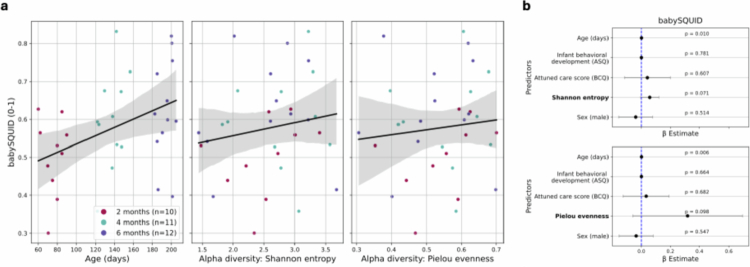
Sleep quality (BabySQUID) in infants. Alpha diversity measured by Shannon entropy and Pielou evenness. (a) Regression lines (95% confidence intervals) of sleep quality along alpha diversity metrics. (b) Coefficient plots. Significant associations between age and the BabySQUID (*p* < 0.05); insignificant associations between Shannon entropy and Pielou evenness, and the BabySQUID (*p* < 0.1). Dots are the coefficients and error bars represent the 95% confidence intervals.

**Figure 6.**
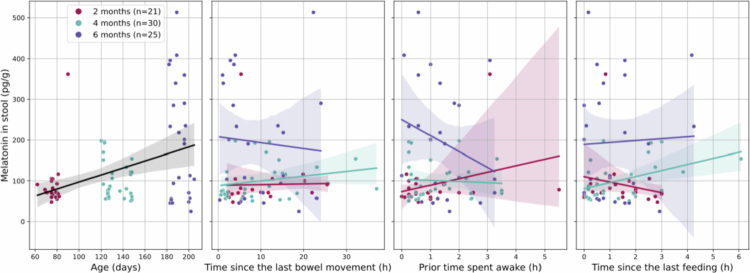
Factors affecting gut melatonin in infants. Regression lines (95% confidence intervals) of melatonin abundance along age (*p* < 0.001), and along the time since the last bowel movement, the prior time spent awake of the infant, and the time since the last feeding (*p* > 0.05).

### Diurnal patterns along age

#### Gut microbiota rhythmicity and sleep rhythmicity

First, to examine possible diurnal links between the gut microbiota and sleep, we tested whether (a) bacterial alpha diversity, or (b) key bacterial genera exhibit diurnal rhythmicity and (c) their associations with sleep rhythmicity. Alpha diversity rhythmicity was assessed in 21 infant-age combinations (Supplementary Figure 9; Equation (3)). The estimates of the cosine fits (*R*^2^ scores) at each age for each diversity metric are displayed in Table 1. For 15 infants, the objective sleep rhythmicity information derived from actimetry were available, with a total of 25 CFIs estimated along all ages, based on actimetry recordings with a median duration of 8 d (std = 2.71, range: 3–11 d) (Table 1; Equation (2)).

In infants with both alpha diversity rhythmicity and sleep rhythmicity assessments (14 infant–age combinations; Equation (3)), no significant association was detected between the two after adjusting for the infants' repeated measures, feeding rhythmicity, parenting style, age and sex (*p* > 0.05, Supplementary Figure 10). Alpha diversity rhythmicity and sleep rhythmicity were also not significantly associated with age after adjusting for repeated measures in the infants and the sex (*p* > 0.05, Supplementary Figure 11). Analysis of age, alpha diversity rhythmicity, and sleep rhythmicity yielded non-significant results, suggesting that these variables were not robustly linked in this cohort (Figure 2, Supplementary Figures 10 and 11).

Exploring rhythmicity in the five most abundant individual genera (Supplementary Figure 3)—*Veillonella*, *Escherichia-Shigella*, *Bifidobacterium*, *Bacteroides* and *Clostridium sensu stricto 1*—(14 infant-age combinations; Equation (3)), no significant associations were detected between genus rhythmicity and sleep rhythmicity, after adjusting for the infants' repeated measures, feeding rhythmicity, parenting style, age and sex, also suggesting that these variables were not robustly linked in this cohort (*p* > 0.05, Figure 2, Supplementary Figures 12 and 13). Still, *Bacteroidetes* rhythmicity showed a weak (insignificant) positive association with age (*p* < 0.1, Supplementary Figure 13; Figure 2a).

#### Microbial temporal volatility and age

As the gut microbiota exhibited only weak rhythmicity, and no clear associations with sleep metrics—based on only 14 infant-age combinations—we hypothesized that bacterial community temporal stability (or its inverse, volatility) could be an alternative marker of microbial community development with age that could relate to sleep activity. An indicator for bacterial community instability, gut microbiota temporal volatility was estimated in all 19 infants at each age (37 data points; 4 cases were excluded (only 1 sample)) (Table 1, Supplementary Figure 14a). There was weak (insignificant) evidence that older infants had lower microbiota temporal volatility, especially when measured using unweighted UniFrac distance, a measure of the fraction of unique branch length, with β_age_ = −2.30 × 10^−4^, SE = 1.37 × 10^−4^, *p* < 0.1 (Figure 3a, Supplementary Figure 14b). In other words, as infants grow up, their microbiota become less volatile over 48 h (i.e., more stable). Female infants had less volatile (i.e., more stable) gut microbiota, when adjusting for the infants' repeated measures and age (β_male_ = 0.0398, SE = 0.0175, *p* < 0.05, Supplementary Figure 14b (Temporal volatility: Bray‒Curtis dissimilarity)).

As microbiota volatility appears to be a useful metric of microbiota development over age, we use this metric alongside alpha diversity in the following analyses as useful metrics for relating gut microbiota stability and diversity to sleep characteristics.

### Modulation of infant sleep by the gut microbiota

#### Sleep rhythmicity: CFI

Next, we investigated the associations among bacterial alpha diversity, temporal volatility, and sleep rhythmicity (*n* = 25; Equation (4)). We observed higher feature richness (β_observed features_ = 6.87 × 10^−3^, SE = 2.54 × 10^−3^, *p* < 0.01) and microbial biodiversity (β_Faith's phylogenetic diversity_ = 0.0721, SE = 0.0337, *p* < 0.05) in infants with increased CFI (Figure 4) when controlling for confounders. No associations were detected for Shannon entropy and Pielou evenness (*p* > 0.05, Supplementary Figure 15). In contrast, there was no association between gut microbiota temporal volatility (bacterial community instability in an infant) and sleep rhythmicity (CFI) (*n* = 23; *p* > 0.05, Supplementary Figure 16; Equation (5)).

Further, the relevance of the parenting context was revealed, such that in several models (alpha diversity: observed features and Faith phylogenetic diversity; temporal volatility: Bray‒Curtis dissimilarity and Jaccard similarity), there was significant evidence of a positive association between infants with a more attuned care (β_BCQ_ = 0.125, SE = 0.0601, *p* < 0.05, Figure 4 (observed features)) and stronger sleep rhythmicity, after adjusting for confounders; but no significant evidence for the development score (Figure 4, Supplementary Figure 17). The results suggest that, on top of greater alpha diversity, a more attuned caring style is also associated with advanced sleep rhythmicity.

#### Sleep quality: BabySQUID

As a subjective sleep parameter, the BabySQUID metric (a composite of nighttime sleep duration, sleep onset latency, bedtime, and number of nighttime awakenings) was estimated in 33 cases from 18 infants (7, 7, and 4 infants had data at respectively 1, 2 or 3 ages) (Table 1). These are all infants with ≥ 2 infant sleep BISQ scores (excluding two entry errors: reported sleep duration of 16 h at 2 months and sleep onset latency of 15 h at 2 months). The BabySQUID was positively associated with age in all the models (β_age_ = 1.04 × 10^−3^, SE = 3.79 × 10^−4^, *p* < 0.01, Figure 5 (Pielou evenness)), when adjusting for the infants' repeated measures and the sex (Equation (6)). However, there were no significant associations between alpha diversity and sleep quality (BabySQUID) in the infants (*p* > 0.05, Figure 5, Supplementary Figure 18). No effect of gut microbiota temporal volatility on sleep quality existed in the infants (*n* = 29, Equation (7); *p* > 0.05, Supplementary Figure 19), indicating that the association was primarily driven by age (*p* < 0.001, Supplementary Figure 19). When k-merized, the microbial composition predicted the BabySQUID (sleep quality) with an accuracy of 63.3% (±8.46%) (Supplementary Table 1, Supplementary Figure 20), indicating performance above random chance (weakly moderate performance) and supporting the potential utility of microbiota profiles as predictive markers of sleep quality.

### Exploratory analyses of melatonin, sleep, and feeding history

In contrast to the previous age-level analyses, we conducted a high-resolution investigation at the sample level to examine the immediate associations between sleep, feeding history, gut melatonin and gut microbiota markers.

#### Gut melatonin

As the primary sleep-regulating hormone in the human body, melatonin was measured in stool samples to examine whether its excretion could be associated with sleep. For samples with measured gut melatonin content, the time since the last bowel movement was estimated; note that one melatonin concentration was excluded as an extreme outlier (>8 standard deviations above the mean) (*n*_samples_ = 76 from 17 infants; Table 1, Supplementary Figure 21).

To identify specific microbial drivers associated with melatonin, we performed a differential abundance analysis at the genus and family levels. We identified the genus *Sutterella* (family *Sutterellaceae*) as a key candidate positively associated with gut melatonin levels (β_*Sutterella*_ = 0.0245, SE = 7.11 × 10^−3^, *p* < 0.05, *q* < 0.1, Supplementary Figure 22a), suggesting a specific link between this genus and the melatonin concentration in the infant gut environment. We also note that the genus is negatively associated with age in the samples (β_*age*_ = −0.0295, SE = 0.0101, *p* < 0.01, *q* < 0.1, Supplementary Figure 22b).

Furthermore, when adjusting for infants' repeated measures, age, and sex, gut melatonin concentration increased with age (β_age_ = 0.638, SE = 0.162, *p* < 0.001, Figure 6; Equation (8)). No significant association was detected between melatonin abundance in the stool and the time since the last bowel movement, sleep history, or feeding history (*p* > 0.05, Supplementary Figure 23).

#### Effects of sleep and feeding history on the gut microbiota

A parallel analysis was conducted on 112 samples where complete sleep and feeding history data were available (Table 1, Supplementary Figure 24; Equation (9)). Figure 3b shows the associations between alpha diversity (measured by feature richness) and previous sleep and feeding history in all infants. All four gut microbiota alpha diversity metrics were positively associated with age (β_age_ = 0.101, SE = 0.0172, *p* < 0.001, Supplementary Figure 25; Figure 3b), when adjusting for the infants' repeated measures and sex. However, alpha diversity was associated with neither sleep nor feeding history (*p* > 0.05, Supplementary Figure 25b; Figure 3b).

## Discussion

To examine associations between the gut microbiota and sleep rhythms in a vulnerable developmental period of infancy, this study combined two methodological strengths: (1) longitudinal within-subject assessments at 2, 4, and 6 months of age combined with (2) high-frequency sampling and continuous monitoring over a 48-h period at each age. We used 16S rRNA gene sequencing to examine the gut microbiota, including diversity, rhythmicity, and temporal volatility patterns, and combined objective actimetry-based measures of sleep rhythmicity with subjective infant sleep metrics (24-h diaries and questionnaires) alongside stool melatonin measurements. As hypothesized, findings revealed rhythmicity patterns within a daily timescale, i.e., diurnality, in the gut microbiota in some infants. However, neither these patterns nor temporal volatility—defined as microbiota variability over 48 h—were associated with sleep rhythmicity, as quantified by a proxy of circadian rhythm (CFI). Infants with higher alpha diversity exhibited stronger sleep rhythmicity (CFI), yet age was the only factor affecting sleep quality (BabySQUID). While microbial diversity and gut melatonin levels were both positively associated with age, they were not affected by the infant's directly preceding sleep or feeding history. The genus *Sutterella* was negatively correlated with age and positively associated with gut melatonin levels in the infants. These findings suggest that the gut microbiome and sleep patterns are linked in early infancy, with several associations observed across age.

Exploring rhythmicity patterns, as expected,^20^^,^^37^ some infants exhibited diurnal rhythms in their gut microbiota, as measured by a cosine fit of alpha diversity and individual genera. However, no associations between microbiota rhythmicity and the infant circadian function index (CFI, sleep rhythmicity) were detected. Still, we report promising (though statistically insignificant) positive associations between gut microbiota rhythmicity and sleep rhythmicity. The findings suggested that rhythmicity patterns—in both the microbiota and sleep—may be modulated by age, with positive (insignificant) associations. The observed increase in microbial rhythmicity with age aligns with previous evidence showing a rise in rhythmic bacterial taxa during the first year of life^37^; specifically, this age-related increase was evident for *Bacteroides*, whereas *Bifidobacterium* did not follow this rhythmic maturation trend in our sample. This also supports the use of the CFI as a reliable proxy for circadian maturation as early as infancy.^20^

Consistent with expectations, findings show an encouraging (though statistically insignificant) decrease in gut microbiota temporal volatility with age. This suggests that as infants grow older, multiple samples collected from the same infant at a given age become more similar to one another (i.e., increased gut bacterial community stability). Microbiota temporal volatility is a novel marker introduced in this study. We consider it a promising complement to traditional diversity measures, providing additional insights into microbiome stability during early development.

Investigating the links between diurnal rhythmicity patterns in the gut microbiota and infant sleep rhythmicity, higher gut bacterial diversities were associated with stronger sleep rhythmicity (as measured by the CFI) in the infants. Notably, we observed higher gut bacterial richness and microbial biodiversity in infants with a stronger sleep rhythmicity, when controlling for confounders. This finding expands on and supports the results from previous studies,^10^^,^^20^^,^^22^ adding a focus on the association between infant gut microbial diversity and sleep rhythmicity. However, this pattern contrasts with findings in other domains of early development, where lower microbial diversity has been associated with more favorable outcomes.^63^^,^^64^ This highlights the need for more nuanced modeling approaches that account for non-linear maturation trajectories, age-specific effects, and potentially even group-level differences. Surprisingly, temporal volatility in the gut microbiota (over 48 h) was not associated with differences in sleep rhythmicity. By quantifying short-term fluctuations in microbiota composition, this approach aimed to provide a high-resolution view of microbial dynamics in relation to behavioral rhythms such as sleep, yet no significant association was observed in this sample.

We found no evidence of an association between microbiota diversity and sleep quality, as defined by the BabySQUID (combining infant sleep variables from the parent-reported BISQ). While studies have investigated the effect of sleep patterns (e.g. evening bedtime) on microbial diversity at a later age,^65^ none have replicated our analysis. Gut microbiota temporal volatility was also not a marker for better sleep quality in this study, and further research should be performed to investigate the associations between gut temporal volatility and sleep quality. Still, the positive associations between age and the BabySQUID strengthen our confidence in the potential utility of this sleep metric in assessing sleep quality in infancy. The random forest classifiers predicted sleep quality (BabySQUID) with moderate accuracy, achieving the best performance and lowest variability when using the top 20 k-mers. As k-mers capture pseudo-phylogenetic sequence-level patterns beyond taxonomic resolution,^59^ this indicates that a targeted subset of microbial features—rather than reductive metrics such as alpha diversity—may carry the most predictive power of sleep quality in the infants. This suggests that the gut microbiota composition, rather than diversity alone, could be a marker for infant sleep quality.

Healthy infants are typically characterized by a low gut bacterial diversity at birth (dominance of *Bifidobacterium*, especially in breast-fed infants),^12^ with increasing richness and diversity over time, reaching an adult-like diversity at 14–24 months of age.^66^^,^^67^ In alignment with this, age was the strongest predictor of gut microbiota diversity in our data.^4^^,^^37^ The influence of the immediate sleep or feeding history context was limited in this study, such that the prior time spent awake, last sleep duration and last feeding time had no effect on microbiota diversity. An explanation could be that in infants the diet type possibly outweighs the effect of fasting (i.e., time since last feeding) on microbial diversity, in contrast to adults.^68^ Breast milk directly transmits bacteria and HMOs from the mother to the infant,^69^ leading to major differences in gut microbiome composition of (exclusively) breast fed and formula fed infants.^4^ In addition, recent evidence highlights chrono-nutritional properties of breast milk, such as circadian fluctuations in hormones and nutrients, which may modulate the infant gut microbiota in breast-fed infants, potentially contributing to the regulation of infants' sleep–wake cycles.^70^ The relationship between sleep recency and duration with alpha diversity in infants is emerging and further studies should be done to properly understand the results.^71^ While studies in adults and animal models have already demonstrated associations between gut microbiota composition and sleep patterns, there is further need for well-controlled interventional trials in infancy that can test causal pathways in the gut-brain-sleep axis.

An exploration of factors affecting gut melatonin in the samples revealed that its abundance increased with age in the infants, in line with previous findings.^22^ Accumulation may result from either the infants' own system or from maternal sources. Endogenous production via the pineal gland is more likely in older infants, although accumulated melatonin from maternal milk may also contribute.^72^ Still, in older infants, we observed a (statistically insignificant) decrease in melatonin levels with increasing time since the last bowel movement and with longer periods spent awake. We previously observed (insignificant) decreases in species diversity with increasing time since the last sleep and feeding at 6 months. This suggests that bacterial metabolic activity could be elevated shortly after these events and then gradually decline over time. We identified the genus *Sutterella* as a key microbial correlate of gut melatonin, though its abundance decreased as infants age, suggesting a specific but transient role for this taxon in the early-life neuroendocrine environment.

Investigating other potential factors influencing infant sleep, we detected that in addition to greater alpha diversity, a more attuned caring style was also associated with more robust sleep rhythmicity. This suggests that the infants' environment is also important in shaping their sleep. Interestingly, after adjusting for age, microbiota temporal variability (a measure of community instability) was lower in females, suggesting that biological sex, along with feeding and environmental factors, might influence microbiota development.^4^ Associations between microbiota development and biological sex have not been reported or found insignificant in most prior studies of term infants,^66^ but have been reported as significant in a longitudinal study of pre-term infants^73^ and could warrant further investigation.

This study represents a pioneering effort in understanding the associations between the gut microbiota, sleep and feeding patterns in infants, by means of high-resolution, continuous sampling and integrative analysis across objective and subjective infant data. The strengths of this pilot study include continuous sampling (*n*_samples_ = 163) with good sequencing resolution, with alpha rarefaction curves (Shannon index) demonstrating near-asymptotic diversity at the minimum sequencing depth of 3035 reads per sample (Supplementary Figure 26). Specifically, high-frequency sampling (median = 4 samples per infant per age (std = 2.45, min = 1, max = 11)) is unique to this investigation, standing in contrast to cross-sectional or sparse longitudinal designs (one sample per infant per age) assessed in prior studies. Multiple samples per age allow for a unique look at diurnal rhythms of the gut microbiota with reduced variability (providing low variability in the results) while accounting for individual differences. Moreover, this study used objective and subjective dimensions of sleep: actimetry, 24-h-diaries, and the BISQ. The objective sleep data came from continuous measures (wearable tracking) averaged over several days (CFI) as a proxy for circadian rhythm in the infant. Finally, all analyses were rigorously controlled for potential confounders (age, sex, feeding history, parenting style and infant behavioral developmental stage), adding some robustness in the results.

To further clarify the relationship between the microbiome and sleep in infancy, larger studies with longer follow-up periods are needed to capture even broader individual developmental trajectories, microbiome maturation patterns, and potential causal directions in the association between gut composition and sleep regulation. In this study, estimating gut microbiota diversity rhythmicity was only possible in 16 infants at a specific age (i.e., age where at least 4 samples were collected within a 48-h period). The low sample size is a result of the multi-measure and demanding continuous monitoring protocol required but reduces the statistical power to detect potential rhythmicity associations (type 2 error). Moreover, this study used objective sleep data as a proxy for circadian rhythm in the infant, unlike previous approaches.^37^ The rhythmicity analysis should be replicated in a study with a larger sample size to confirm our promising findings. Analyses on specific taxa (e.g., *Bifidobacterium*, major metabolizers of HMOs) could also be investigated in larger studies to provide explanations for the mechanistic pathways involved. Future randomized control studies should investigate causal mechanisms, test these relationships in more diverse or at-risk populations, and explore the long-term implications for infant (neuro)development.^71^ Moreover, while the BabySQUID provides a useful pioneer approach to condensing multidimensional sleep data into a single proxy, we acknowledge that the relative contribution of each parameter remains complex; specifically, a fast sleep onset latency could be indicative of an overall lack of sleep, while earlier bedtimes may not generally reflect infants with better sleep quality. Future research could further refine this by employing computational approaches to determine the respective weights of individual variables, similar to previous composite sleep modeling approaches.^7^ In addition, we expect infant sleep to be influenced by the real-life context or cultural/social circumstances, including factors that were not monitored in this study. To continue, any apparent relationships between microbiome features and sleep patterns in this study could be confounded by dietary changes over the study period (e.g., when infants transitioned to formula and solid food), untracked in this study. Finally, the microbiome‒sleep relationship appears to be bidirectional, which could confound interpretations of the present findings and highlight the need for longitudinal or interventional studies to disentangle the directionality of this relationship.^5^

This study highlights the intricate relationships among gut microbiota rhythmicity, diversity and temporal volatility, with infant sleep rhythmicity and quality. It also integrates information on feeding patterns, parenting care style, behavior development, and gut melatonin. With both a diurnal and longitudinal resolution, this study provides a foundation for future research, offering insights into the potential role of the gut microbiota in shaping sleep rhythmicity and sleep consolidation in infants. Looking ahead, these findings open up promising avenues for targeted interventions—ranging from dietary strategies to microbiota-based therapies—that could support healthy sleep and (neuro)development in infants.

## Supplementary Material

Supplementary material Supplementary Figures and Tables.

## Data Availability

Data and code availability Newly generated sequencing data (16S rRNA) from stool samples in this study are publicly available in the NCBI Sequence Read Archive (SRA) under accession code PRJEB104111. All original code supporting the findings of this study are publicly available on GitHub at: https://github.com/bokulich-publications/microbiome-sleep-rhythmicity Any additional information required to reanalyze the data reported in this paper is available from the corresponding authors upon request.

## References

[1] Bokulich NA, Chung J, Battaglia T, Henderson N, Jay M, Li H, Lieber A, Wu F, Perez-Perez GI, Chen Y, et al. Antibiotics, birth mode, and diet shape microbiome maturation during early life. Sci Transl Med. 2016;8:343ra8–343ra8. doi: 10.1126/scitranslmed.aad7121. PMC5308924 27306664

[2] Fahur Bottino G, Bonham KS, Patel F, McCann S, Zieff M, Naspolini N, Ho D, Portlock T, Joos R, Midani FS, et al. Early life microbial succession in the gut follows common patterns in humans across the globe. Nat Commun. 2025;16:660. doi: 10.1038/s41467-025-56072-w. 39809768 PMC11733223

[3] La Rosa PS, Warner BB, Zhou Y, Weinstock GM, Sodergren E, Hall-Moore CM, Stevens HJ, Bennett WE, Shaikh N, Linneman LA, et al. Patterned progression of bacterial populations in the premature infant gut. Proc Natl Acad Sci U S A. 2014;111:12522–12527. doi: 10.1073/pnas.1409497111. 25114261 PMC4151715

[4] Robertson RC, Manges AR, Finlay BB, Prendergast AJ. The human microbiome and child growth – first 1000 days and beyond. Trends Microbiol. 2019;27:131–147. doi: 10.1016/j.tim.2018.09.008. 30529020

[5] Sen P, Molinero-Perez A, O'Riordan KJ, McCafferty CP, O'Halloran KD, Cryan JF. Microbiota and sleep: awakening the gut feeling. Trends Mol Med. 2021;27:935–945. doi: 10.1016/j.molmed.2021.07.004. 34364787

[6] Ahrens AP, Hyötyläinen T, Petrone JR, Igelström K, George CD, Garrett TJ, Orešič M, Triplett EW, Ludvigsson J. Infant microbes and metabolites point to childhood neurodevelopmental disorders. Cell. 2024;187:1853–1873.e15. doi: 10.1016/j.cell.2024.02.035. 38574728

[7] Schoch SF, Huber R, Kohler M, Kurth S. Which are the central aspects of infant sleep? The dynamics of sleep composites across infancy. Sensors. 2020;20:7188. doi: 10.3390/s20247188. 33333904 PMC7765288

[8] Meltzer LJ, Williamson AA, Mindell JA. Pediatric sleep health: it matters, and so does how we define it. Sleep Med Rev. 2021;57:101425. doi: 10.1016/j.smrv.2021.101425. 33601324 PMC9067252

[9] Timofeev I, Schoch SF, LeBourgeois MK, Huber R, Riedner BA, Kurth S. Spatio-temporal properties of sleep slow waves and implications for development. Curr Opin Physiol. 2020;15:172–182. doi: 10.1016/j.cophys.2020.01.007. 32455180 PMC7243595

[10] Schoch SF, Castro-Mejía J, Krych L, Leng B, Kot W, Kohler M, Huber R, Rogler G, Biedermann L, Walser J, et al. From alpha diversity to zzz: interactions among sleep, the brain, and gut microbiota in the first year of life. Prog Neurobiol. 2022;209:102208. doi: 10.1016/j.pneurobio.2021.102208. 34923049

[11] Schoch SF, Jaramillo V, Markovic A, Huber R, Kohler M, Jenni OG, Lustenberger C, Kurth S. Bedtime to the brain: how infants' sleep behaviours intertwine with non-rapid eye movement sleep electroencephalography features. J Sleep Res. 2024;33:e13936. doi: 10.1111/jsr.13936. 37217191

[12] Lawson MAE, O'Neill IJ, Kujawska M, Gowrinadh Javvadi S, Wijeyesekera A, Flegg Z, Chalklen L, Hall LJ. Breast milk-derived human milk oligosaccharides promote bifidobacterium interactions within a single ecosystem. ISME J. 2020;14:635–648. doi: 10.1038/s41396-019-0553-2. 31740752 PMC6976680

[13] Wiciński M, Sawicka E, Gębalski J, Kubiak K, Malinowski B. Human milk oligosaccharides: health benefits, potential applications in infant formulas, and pharmacology. Nutrients. 2020;12:266. doi: 10.3390/nu12010266. 31968617 PMC7019891

[14] Heath A-LM, Haszard JJ, Galland BC, Lawley B, Rehrer NJ, Drummond LN, Sims IM, Taylor RW, Otal A, Tannock GW. Association between the faecal short-chain fatty acid propionate and infant sleep. Eur J Clin Nutr. 2020;74:1362–1365. doi: 10.1038/s41430-019-0556-0. 31969698

[15] Colombo J, Carlson SE, Algarín C, Reyes S, Chichlowski M, Harris CL, Wampler JL, Peirano P, Berseth CL. Developmental effects on sleep–wake patterns in infants receiving a cow's milk-based infant formula with an added prebiotic blend: a randomized controlled trial. Pediatr Res. 2021;89:1222–1231. doi: 10.1038/s41390-020-1044-x. 32615579 PMC8119237

[16] Nocerino R, De Filippis F, Cecere G, Marino A, Micillo M, Di Scala C, de Caro C, Calignano A, Bruno C, Paparo L, et al. The therapeutic efficacy of *Bifidobacterium animalis* subsp. *lactis* BB-12® in infant colic: a randomised, double blind, placebo-controlled trial. Aliment Pharmacol Ther. 2020;51:110–120. doi: 10.1111/apt.15561. 31797399 PMC6973258

[17] Sung V, Hiscock H, Tang MLK, Mensah FK, Nation ML, Satzke C, Heine RG, Stock A, Barr RG, Wake M. Treating infant colic with the probiotic *Lactobacillus reuteri*: double blind, placebo controlled randomised trial. BMJ. 2014;348: g2107. 10.1136/bmj.g2107. 24690625 PMC3972414

[18] Papagaroufalis K, Fotiou A, Egli D, Tran L-A, Steenhout P. A randomized double blind controlled safety trial evaluating D-Lactic acid production in healthy infants fed a *Lactobacillus reuteri*-containing formula. Nutr Metab Insights. 2014;7:19–27. doi: 10.4137/NMI.S14113. 24812520 PMC3999946

[19] Mühlematter C, Nielsen DS, Castro-Mejía JL, Brown SA, Rasch B, Wright KP, Walser J, Schoch SF, Kurth S, Nakamura TJ. Not simply a matter of parents—infants' sleep-wake patterns are associated with their regularity of eating. PLoS One. 2023;18:e0291441. doi: 10.1371/journal.pone.0291441. 37796923 PMC10553286

[20] Mühlematter C, Nielsen DS, Castro-Mejía JL, Walser J-C, Schoch SF, Kurth S. Rhythms of early life: gut microbiota rhythmicity and circadian maturation in infants. bioRxiv. 2025. doi: 10.1101/2025.01.06.631487. PMC12644702 41285943

[21] Kaczmarek JL, Musaad SM, Holscher HD. Time of day and eating behaviors are associated with the composition and function of the human gastrointestinal microbiota. Am J Clin Nutr. 2017;106:1220–1231. doi: 10.3945/ajcn.117.156380. 28971851

[22] Kurth S, Al-Andoli M, Zimmermann P, Schoch S, Markovic A, Mühlematter C, Beaugrand M, Jenni O, Liamlahi R, Walser J-C, Nielsen D. Fecal melatonin as a biomarker of emerging circadian maturity and gut microbiota in infancy. Research Square. 2025. doi:10.21203/rs.3.rs-6569729/v1. PMC13125696 42050171

[23] Zimmermann P., Kurth S., Pugin B., Bokulich N. A. Microbial melatonin metabolism in the human intestine as a therapeutic target for dysbiosis and rhythm disorders. npj Biofilms Microbiomes. 2024;10:1–17. 39604427 10.1038/s41522-024-00605-6 PMC11603051

[24] Meng X, Li Y, Zhou Y, Gan R, Xu D. Dietary sources and bioactivities of melatonin. Nutrients. 2017;9:367. doi: 10.3390/nu9040367. 28387721 PMC5409706

[25] Attanasio A, Rager K, Gupta D. Ontogeny of circadian rhythmicity for melatonin, serotonin, and N-acetylserotonin in humans. J Pineal Res. 1986;3:251–256. doi: 10.1111/j.1600-079X.1986.tb00747.x. 3772723

[26] Kennaway DJ, Goble FC, Stamp GE. Factors influencing the development of melatonin rhythmicity in humans. J Clin Endocrinol Metab. 1996;81:1525–1532. 8636362 10.1210/jcem.81.4.8636362

[27] Gao T, Wang Z, Dong Y, Cao J, Chen Y. Butyrate ameliorates insufficient sleep-induced intestinal mucosal damage in humans and mice. Microbiol Spectr. 2022;11:e02000–22. 36541814 10.1128/spectrum.02000-22 PMC9927315

[28] Grosicki GJ, Riemann BL, Flatt AA, Valentino T, Lustgarten MS. Self-reported sleep quality is associated with gut microbiome composition in young, healthy individuals: a pilot study. Sleep Med. 2020;73:76–81. doi: 10.1016/j.sleep.2020.04.013. 32795890 PMC7487045

[29] Smith RP, Easson C, Lyle SM, Kapoor R, Donnelly CP, Davidson EJ, Parikh E, Lopez JV, Tartar JL, Aich P. Gut microbiome diversity is associated with sleep physiology in humans. PLoS One. 2019;14:e0222394. doi: 10.1371/journal.pone.0222394. 31589627 PMC6779243

[30] Liu B, Lin W, Chen S, Xiang T, Yang Y, Yin Y, Xu G, Pan J, Xie L. Gut microbiota as an objective measurement for auxiliary diagnosis of insomnia disorder. Front Microbiol. 2019;10:1770. doi: 10.3389/fmicb.2019.01770. 31456757 PMC6701205

[31] Deng W, Wang S, Li F, Xing YP, Lv Y, Ke H, Hao H, Chen Y, Xiao X. Gastrointestinal symptoms have a minor impact on autism spectrum disorder and associations with gut microbiota and short-chain fatty acids. Front Microbiol. 2022;13:1000419. doi: 10.3389/fmicb.2022.1000419. 36274684 PMC9585932

[32] Hua X, Zhu J, Yang T, Guo M, Li Q, Chen J. The gut microbiota and associated metabolites are altered in sleep disorder of children with autism spectrum disorders. Front Psychiatry. 2020;11:855. doi: 10.3389/fpsyt.2020.00855. 32982808 PMC7493623

[33] Lawrence K, Myrissa K, Toribio-Mateas M, Minini L, Gregory AM. Trialling a microbiome-targeted dietary intervention in children with ADHD—the rationale and a non-randomised feasibility study. Pilot Feasibility Stud. 2022;8:108. doi: 10.1186/s40814-022-01058-4. 35606889 PMC9125862

[34] Valentini F, Evangelisti M, Arpinelli M, Di Nardo G, Borro M, Simmaco M, Villa MP. Gut microbiota composition in children with obstructive sleep apnoea syndrome: a pilot study. Sleep Med. 2020;76:140–147. doi: 10.1016/j.sleep.2020.10.017. 33181474

[35] Wang Y, van de Wouw M, Drogos L, Vaghef-Mehrabani E, Reimer RA, Tomfohr-Madsen L, Giesbrecht GF. Sleep and the gut microbiota in preschool-aged children. Sleep. 2022;45:zsac020. doi: 10.1093/sleep/zsac020. 35037059 PMC9189981

[36] Xiang X, Chen J, Zhu M, Gao H, Liu X, Wang Q. Multiomics revealed the multi-dimensional effects of late sleep on gut microbiota and metabolites in children in northwest China. Nutrients. 2023;15:4315. doi: 10.3390/nu15204315. 37892391 PMC10609417

[37] Heppner N, Reitmeier S, Heddes M, Merino MV, Schwartz L, Dietrich A, List M, Gigl M, Meng C, van der Veen DR, et al. Diurnal rhythmicity of infant fecal microbiota and metabolites: a randomized controlled interventional trial with infant formula. Cell Host Microbe. 2024;32:573–587.e5. doi: 10.1016/j.chom.2024.02.015. 38569545

[38] Werner H, Molinari L, Guyer C, Jenni OG. Agreement rates between actigraphy, diary, and questionnaire for children's sleep patterns. Arch Pediatr Adolesc Med. 2008;162:350–358. doi: 10.1001/archpedi.162.4.350. 18391144

[39] Schoch SF, Jenni OG, Kohler M, Kurth S. Actimetry in infant sleep research: an approach to facilitate comparability. Sleep. 2019;42:zsz083. doi: 10.1093/sleep/zsz083. 30941431 PMC6612674

[40] Sadeh A. A brief screening questionnaire for infant sleep problems: validation and findings for an Internet sample. Pediatrics. 2004;113:e570–e577. doi: 10.1542/peds.113.6.e570. 15173539

[41] Winstanley A, Gattis M. The baby care questionnaire: a measure of parenting principles and practices during infancy. Infant Behav Dev. 2013;36:762–775. doi: 10.1016/j.infbeh.2013.08.004. 24050932 PMC3878760

[42] Squires J, Potter J, Bricker L. The ASQ user's guide for the ages & stages questionnaires: a parent-completed, child-monitoring system. 1995. Baltimore, MD, US: Paul H Brookes Publishing. p. 156.

[43] Flörl L, Cabrera PM, Moccia MD, Plüss S, Bokulich NA. HighALPS: ultra-high-throughput marker-gene amplicon library preparation and sequencing on the illumina NextSeq and NovaSeq platforms. mSystems. 2026;11:e00023-26. doi: 10.1128/msystems.00023-26. 41665368 PMC13011336

[44] Apprill A, McNally S, Parsons R, Weber L. Minor revision to V4 region SSU rRNA 806R gene primer greatly increases detection of SAR11 bacterioplankton. Aquat Microb Ecol. 2015;75:129–137. doi: 10.3354/ame01753.

[45] Parada AE, Needham DM, Fuhrman JA. Every base matters: assessing small subunit rRNA primers for marine microbiomes with mock communities, time series and global field samples. Environ Microbiol. 2016;18:1403–1414. doi: 10.1111/1462-2920.13023. 26271760

[46] Bolyen E, Rideout JR, Dillon MR, Bokulich NA, Abnet CC, Al-Ghalith GA, Alexander H, Alm EJ, Arumugam M, Asnicar F, et al. Reproducible, interactive, scalable and extensible microbiome data science using QIIME 2. Nat Biotechnol. 2019;37:852–857. doi: 10.1038/s41587-019-0209-9. 31341288 PMC7015180

[47] Callahan BJ, McMurdie PJ, Rosen MJ, Han AW, Johnson AJA, Holmes SP. DADA2: high-resolution sample inference from illumina amplicon data. Nat Methods. 2016;13:581–583. doi: 10.1038/nmeth.3869. 27214047 PMC4927377

[48] Bokulich NA, Kaehler BD, Rideout JR, Dillon M, Bolyen E, Knight R, Huttley GA, Gregory Caporaso J. Optimizing taxonomic classification of marker-gene amplicon sequences with QIIME 2's q2-feature-classifier plugin. Microbiome. 2018;6:90. doi: 10.1186/s40168-018-0470-z. 29773078 PMC5956843

[49] Pruesse E, Quast C, Knittel K, Fuchs BM, Ludwig W, Peplies J, Glockner FO. SILVA: a comprehensive online resource for quality checked and aligned ribosomal RNA sequence data compatible with ARB. Nucleic Acids Res. 2007;35:7188–7196. doi: 10.1093/nar/gkm864. 17947321 PMC2175337

[50] Quast C, Pruesse E, Yilmaz P, Gerken J, Schweer T, Yarza P, Peplies J, Glöckner FO. The SILVA ribosomal RNA gene database project: improved data processing and web-based tools. Nucleic Acids Res. 2013;41:D590–D596. doi: 10.1093/nar/gks1219. 23193283 PMC3531112

[51] Robeson MS, O'Rourke DR, Kaehler BD, Ziemski M, Dillon MR, Foster JT, Bokulich NA, Pertea M. RESCRIPt: reproducible sequence taxonomy reference database management. PLoS Comput Biol. 2021;17:e1009581. doi: 10.1371/journal.pcbi.1009581. 34748542 PMC8601625

[52] Raspet I, Gehret E, Herman C, Meilander J, Manley A, Simard A, Bolyen E, Caporaso JC. Facilitating bootstrapped and rarefaction-based microbiome diversity analysis with q2-boots. arXiv. 2024 doi:10.48550/arXiv.2408.05420. PMC12489448 41049561

[53] Sørensen T, Sørensen T, Biering-Sørensen T, Sørensen T, Sorensen JT. A method of establishing group of equal amplitude in plant sociobiology based on similarity of species content and its application to analyses of the vegetation on Danish commons. Biol Skr. 1948;5:1–34.

[54] Jaccard P. Nouvelles recherches sur la distribution florale. Bull Soc Vaud Sci Nat. 1908;44(163):223–270. doi: 10.5169/seals-268384.

[55] Lozupone C, Knight R. UniFrac: a new phylogenetic method for comparing microbial communities. Appl Environ Microbiol. 2005;71:8228–8235. doi: 10.1128/AEM.71.12.8228-8235.2005. 16332807 PMC1317376

[56] Lozupone CA, Hamady M, Kelley ST, Knight R. Quantitative and qualitative β diversity measures lead to different insights into factors that structure microbial communities. Appl Environ Microbiol. 2007;73:1576–1585. doi: 10.1128/AEM.01996-06. 17220268 PMC1828774

[57] Markovic A, Mühlematter C, Blume C, Zimmermann P, Kurth S. From womb to crib: how fetal activity patterns in utero reveal postnatal sleep behavior. bioRxiv. 2024. doi:10.1101/2024.12.04.626754.

[58] Ortiz-Tudela E, Martinez-Nicolas A, Campos M, Rol MÁ, Madrid JA. A new integrated variable based on thermometry, actimetry and body position (TAP) to evaluate circadian system status in humans. PLoS Comput Biol. 2010;6:e1000996. doi: 10.1371/journal.pcbi.1000996. 21085644 PMC2978699

[59] Bokulich NA. Integrating sequence composition information into microbial diversity analyses with k-mer frequency counting. mSystems. 2025. e01550-24. doi: 10.1128/msystems.01550-24. 39976436 PMC11915819

[60] Pedregosa F, Varoquaux G, Gramfort A, Michel V, Thirion B, Grisel O, Blondel M, Prettenhofer P, Weiss R, Dubourg V, Vanderplas J, Passos A, Cournapeau D, Brucher M, Perrot M, Duchesnay E. Scikit-learn: machine learning in python. J Mach Learn Res. 2011;12:2825–2830.

[61] Lin H, Peddada SD. Multigroup analysis of compositions of microbiomes with covariate adjustments and repeated measures. Nat Methods. 2024;21:83–91. doi: 10.1038/s41592-023-02092-7. 38158428 PMC10776411

[62] Benjamini Y, Hochberg Y. Controlling the false discovery rate: a practical and powerful approach to multiple testing. J R Stat Soc B. 1995;57:289–300. doi: 10.1111/j.2517-6161.1995.tb02031.x.

[63] Alderete TL, Jones RB, Shaffer JP, Holzhausen EA, Patterson WB, Kazemian E, Chatzi L, Knight R, Plows JF, Berger PK, et al. Early life gut microbiota is associated with rapid infant growth in Hispanics from Southern California. Gut Microbes. 2021;13:1961203. doi: 10.1080/19490976.2021.1961203. 34424832 PMC8386720

[64] Moroishi Y, Gui J, Hoen AG, Morrison HG, Baker ER, Nadeau KC, Li H, Madan JC, Karagas MR. The relationship between the gut microbiome and the risk of respiratory infections among newborns. Commun Med. 2022;2:87. doi: 10.1038/s43856-022-00152-1. 35847562 PMC9283516

[65] Olson M, Toffoli S, Vander Wyst KB, Zhou F, Reifsnider E, Petrov ME, Whisner CM. Associations of infant feeding, sleep, and weight gain with the toddler gut microbiome. Microorganisms. 2024;12:549. doi: 10.3390/microorganisms12030549. 38543600 PMC10972346

[66] Stewart CJ, Ajami NJ, O'Brien JL, Hutchinson DS, Smith DP, Wong MC, Ross MC, Lloyd RE, Doddapaneni H, Metcalf GA, et al. Temporal development of the gut microbiome in early childhood from the TEDDY study. Nature. 2018;562:583–588. doi: 10.1038/s41586-018-0617-x. 30356187 PMC6415775

[67] Wernroth M-L, Peura S, Hedman AM, Hetty S, Vicenzi S, Kennedy B, Fall K, Svennblad B, Andolf E, Pershagen G, et al. Development of gut microbiota during the first 2 years of life. Sci Rep. 2022;12:9080. doi: 10.1038/s41598-022-13009-3. 35641542 PMC9156670

[68] Zeb F, Wu X, Chen L, Fatima S, Haq I, Majeed F, Feng Q, Li M. Effect of time-restricted feeding on metabolic risk and circadian rhythm associated with gut microbiome in healthy males. Br J Nutr. 2020;123:1216–1226. doi: 10.1017/S0007114519003428. 31902372

[69] Selma-Royo M, Dubois L, Manara S, Armanini F, Cabrera-Rubio R, Valles-Colomer M, González S, Parra-Llorca A, Escuriet R, Bode L, et al. Birthmode and environment-dependent microbiota transmission dynamics are complemented by breastfeeding during the first year. Cell Host Microbe. 2024;32:996–1010. doi: 10.1016/j.chom.2024.05.005. 38870906 PMC11183301

[70] Aksu S, Arslan S. The circadian composition of breast milk: a natural starting point for chrononutrition. Curr Nutr Rep. 2026;15:21. doi: 10.1007/s13668-026-00749-1. 41790368 PMC12966228

[71] Zimmermann P, Kurth S, Giannoukos S, Stocker M, Bokulich NA. NapBiome trial: targeting gut microbiota to improve sleep rhythm and developmental and behavioural outcomes in early childhood in a birth cohort in Switzerland – a study protocol. BMJ Open. 2025;15:e092938. doi: 10.1136/bmjopen-2024-092938. PMC11877202 40032396

[72] Häusler S, Lanzinger E, Sams E, Fazelnia C, Allmer K, Binder C, Reiter RJ, Felder TK. Melatonin in human breast milk and its potential role in circadian entrainment: a nod towards chrononutrition? Nutrients. 2024;16:1422. doi: 10.3390/nu16101422. 38794660 PMC11124029

[73] Chen J, Li H, Hird SM, Xu W, Maas K, Cong X. Sex differences in gut microbial development of preterm infant twins in early life: a longitudinal analysis. Front Cell Infect Microbiol. 2021;11. doi:10.3389/fcimb.2021.671074. PMC8387566 34458157

