# Supplementary material for: The gut microbiota and sleep in infants: a focus on diurnal rhythmicity patterns
Source: Gut Microbes Rep. 2026 Mar 29;3(1):2649096. doi: 10.1080/29933935.2026.2649096 (PMC13037436; doi:10.1080/29933935.2026.2649096)
Supplement: Supplementary material — Supplementary Figures and Tables. [file KGMR_A_2649096_SM2950.docx]

**Supplementary Figures and Tables**

Related to: The gut microbiota and sleep in infants: a focus on diurnal rhythmicity patterns (Kerff et al., 2026)

Suppl. Fig. 1. **Flow chart of the inclusion and exclusion process of samples.**

Suppl. Fig. 2. **Stool samples collection times across all ages and infants**.

Suppl. Fig. 3. **Relative abundance of the infants’ gut microbiota across all ages**. (top) Gut bacterial phyla, and (bottom) gut bacterial genera.


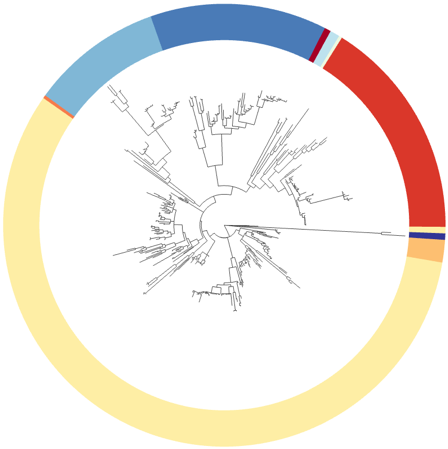

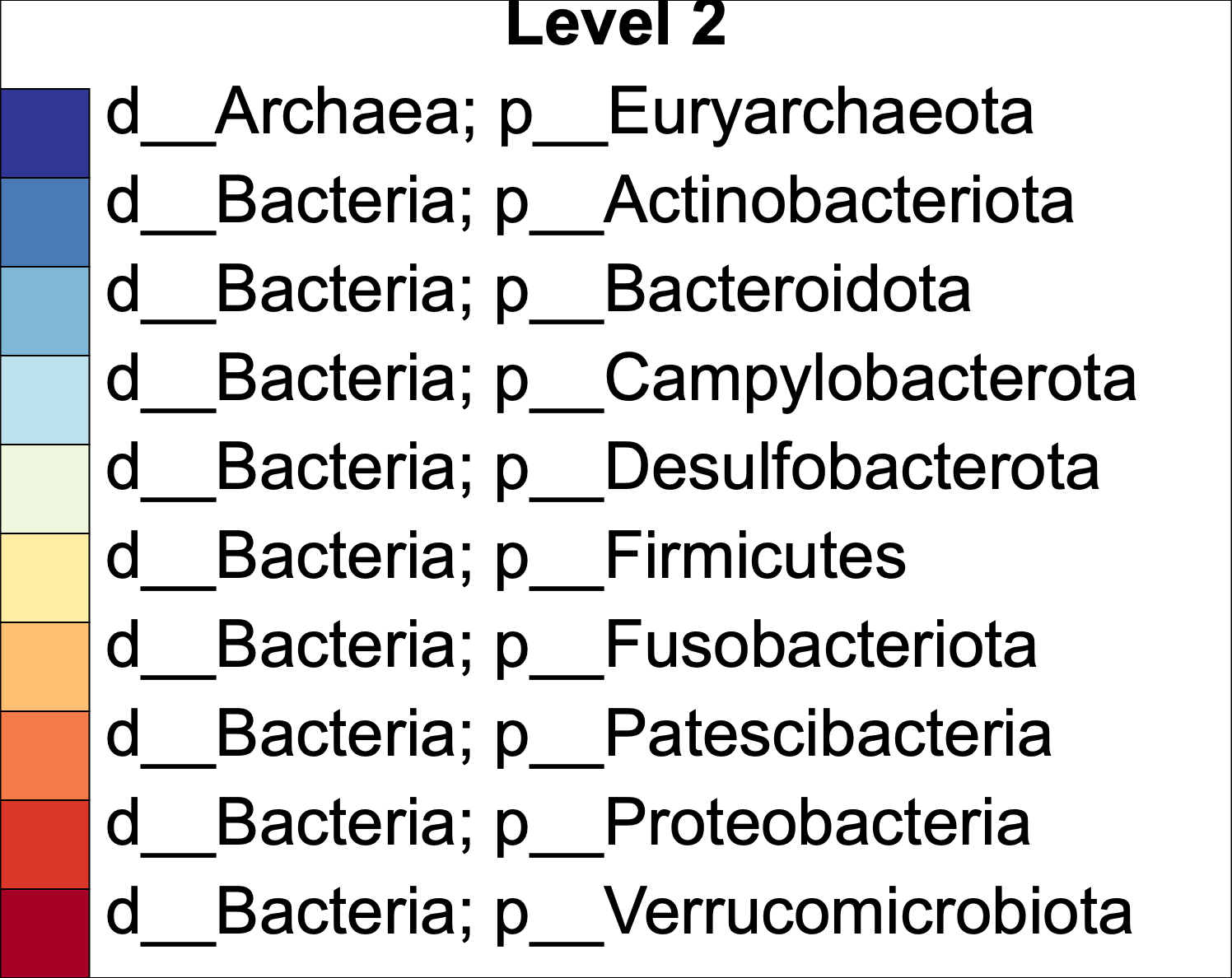


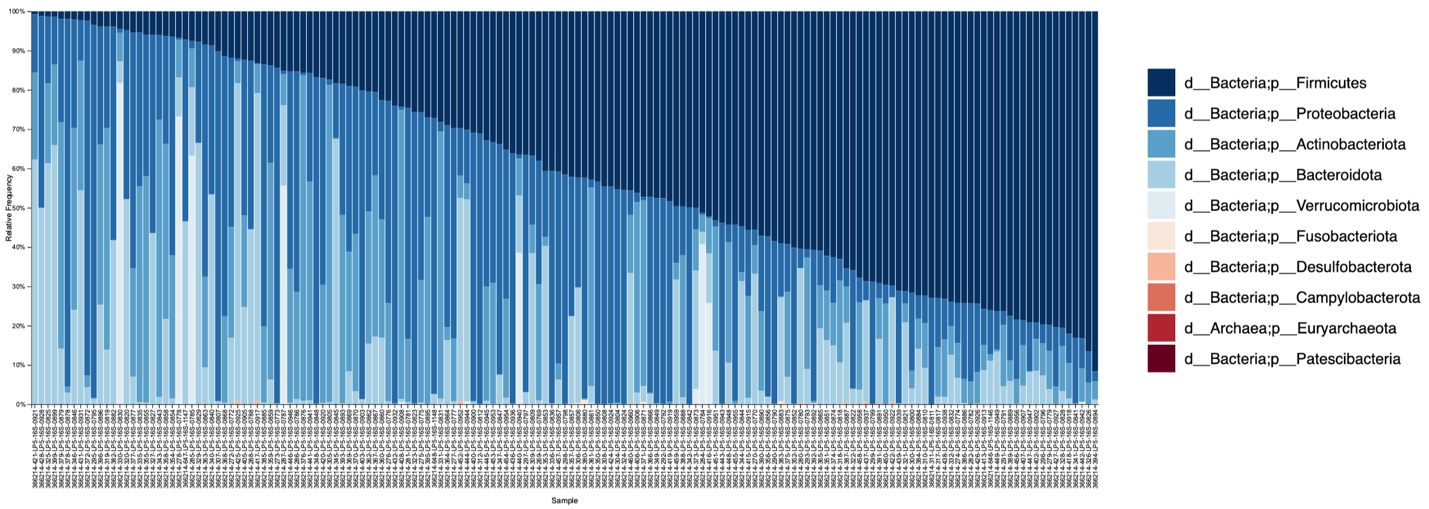


Suppl. Fig. 4. **Gut microbiota composition**. (top) Phylogenetic tree and (bottom) stacked bar plots of the phyla present in the samples.

Suppl. Fig. 5. **Gut microbiota beta diversity (between-samples (dis)similarity)**. Each color is an infant. **a** Features measured using ASVs. Each large circle represents the centroid of an infant; smaller circles represent individual longitudinal samples. **b** Features measured using k-mers.

Suppl. Fig. 6. **Infant sleep** **BISQ variables across all ages.** Sleep duration, sleep onset latency, bedtime and frequency of waking in the night variables.

Suppl. Fig. 7. **BCQ variables across all ages.** Structured and attuned care scores.

Suppl. Fig. 8. **ASQ Composite score across all ages.** Represents communication, fine motor, gross motor, personal-social and problem solving scores.

Suppl. Fig. 9. **Cosine fitting of alpha diversity metrics across age.** Cosine functions are plotted for the infants having at least 4 samples per age.

Suppl. Fig. 10. **Sleep rhythmicity (CFI) and alpha diversity rhythmicity**. Alpha diversity rhythmicity measured by the cosine fit (R^2^ score) of observed features, Shannon entropy, Pielou evenness and Faith's phylogenetic diversity. **a** Regression lines (95% confidence intervals). **b** Coefficient plots. No significant associations (p>0.05; n=14). Dots are the coefficients and error bars represent the 95% confidence intervals.

Suppl. Fig. 11. **Alpha diversity rhythmicity and age**. Alpha diversity rhythmicity measured by the cosine fit (R^2^ score) of observed features, Shannon entropy, Pielou evenness and Faith's phylogenetic diversity (p>0.05). Only infants having at least 4 samples per age are included. **a** Regression lines (95% confidence intervals) of rhythmicity metrics along age. **b** Coefficient plots: age and sex effects on alpha diversity rhythmicity (n=21) and sleep rhythmicity (last sub-figure; n=25). No significant associations (p>0.05). The p-value for age of the fourth model (Faith Phylogenetic diversity) is reported as NaN most probably due to insufficient within-subject variation: 11 infants had only one datapoint, which limits the model’s ability to estimate the within-subject effect of the variables. Dots are the coefficients and error bars represent the 95% confidence intervals.

Suppl. Fig. 12. **Sleep rhythmicity (CFI) and individual genus rhythmicity**. Genera rhythmicity measured by the cosine fit (R^2^ score) of *Veillonella, Bifidobacterium*, *Escherichia-Shigella*, *Bacteroides* and *Clostridium* relative abundances. **a** Regression lines (95% confidence intervals) of genera rhythmicity metrics along sleep rhythmicity. **b** Coefficient plots. No significant associations (p>0.05; n=14). Dots are the coefficients and error bars represent the 95% confidence intervals.

Suppl. Fig. 13 **Individual genus rhythmicity and age**. Genera rhythmicity measured by the cosine fit (R^2^ score) of *Veillonella, Bifidobacterium*, *Escherichia-Shigella*, *Bacteroides* and *Clostridium* relative abundances. Only infants having at least 4 samples per age are included (n=21). **a** Regression lines (95% confidence intervals) of genera rhythmicity metrics along age. **b** Coefficient plots. Limited evidence of an association between the *Bacteroides* rhythmicity and age (p<0.1). Dots are the coefficients and error bars represent the 95% confidence intervals.

Suppl. Fig. 14. **The gut microbiota temporal volatility and age**. Gut microbiota temporal volatility measured by Bray-Curtis dissimilarity, Jaccard similarity index, Unweighted UniFrac and Weighted UniFrac distances. Only cases with at least 2 samples per age are included. **a** Regression lines (95% confidence intervals) of temporal volatility metrics along age; limited evidence of an association between the unweighted UniFrac distance and age (p<0.1). **b** Coefficient plots. Significant association between the temporal volatility and the sex (p<0.05). Dots are the coefficients and error bars represent the 95% confidence intervals.

Suppl. Fig. 15. **Sleep rhythmicity (CFI) and alpha diversity**. Alpha diversity measured by Shannon entropy (features abundances and evenness) and Pielou evenness (features evenness). **a** Regression lines (95% confidence intervals) of sleep rhythmicity along alpha diversity metrics. **b** Coefficient plots. No significant associations (p>0.05). Dots are the coefficients and error bars represent the 95% confidence intervals.

Suppl. Fig. 16. **Sleep rhythmicity (CFI) and gut microbiota temporal volatility**. Gut microbiota temporal volatility measured by Bray-Curtis dissimilarity, Jaccard similarity index, Unweighted UniFrac and Weighted UniFrac distances. **a** Regression lines (95% confidence intervals) of sleep rhythmicity along temporal volatility metrics. **b** Coefficient plots. Significant association between the sleep rhythmicity and the development composite and attuned care scores (p<0.05). Dots are the coefficients and error bars represent the 95% confidence intervals.

Suppl. Fig. 17. **Sleep rhythmicity (CFI) and behavioral development in infants**. Infant behavioral developmental stage measured by the composite score of the ASQ. Regression lines (95% confidence intervals).

Suppl. Fig. 18. **Sleep quality (babySQUID) and alpha diversity**. Alpha diversity measured by observed features (features abundance) and Faith phylogenetic diversity (biodiversity). **a** Regression lines (95% confidence intervals) of sleep quality along alpha diversity. **b** Coefficient plots. Significant associations between age and sleep quality (p<0.05). Dots are the coefficients and error bars represent the 95% confidence intervals.

Suppl. Fig. 19. **Sleep quality (babySQUID) and gut microbiota temporal volatility.** Gut microbiota temporal volatility measured by Bray-Curtis dissimilarity, Jaccard similarity index, Unweighted UniFrac and Weighted UniFrac distances. **a** Regression lines (95% confidence intervals) of sleep quality along temporal volatility. **b** Coefficient plots. Significant associations between age and sleep quality (babySQUID) (p<0.001). Dots are the coefficients and error bars represent the 95% confidence intervals.

Suppl. Fig. 20. **Confusion matrix showing the results of the best random forest classifier**. The model predicts the babySQUID (n = 33) binarized at the median (0 if below median, 1 if > median) using as input the k-merized feature table (k-mer length = 16; top 20 features selected), with a GroupKFold (n=5) cross-validation (K-fold iterator variant with non-overlapping groups). Diagonal = correct predictions (n = 5); Off diagonal = misclassifications (n = 2).

Suppl. Fig. 21. **Gut melatonin and time since the last bowel movement at each stool sample.** Visualization limited to samples with melatonin (n_samples_=125) and previous stool sample (n_samples_=127) information.

Suppl. Fig. 22. **Volcano plots** **of the differentially abundant genus**. The y-axis displays the statistical significance as -log_10_(p-value). Each point represents a unique taxon. Points colored in red represent taxa reaching a false discovery rate (FDR) threshold of q < 0.1, while grey points represent non-significant taxa. The horizontal dashed line indicates the significance threshold (p = 0.05). **a** Melatonin-associated taxa. The x-axis represents the log fold change (LFC) of gut melatonin abundance, where positive values indicate enrichment and negative values indicate depletion in gut melatonin abundance. **b** Age-associated taxa. The x-axis represents the LFC in age.

Suppl. Fig. 23. **Coefficient plot showing factors effects on gut melatonin.** Melatonin abundance positively associated with age (p<0.001). Dots are the coefficients and error bars represent the 95% confidence intervals.

Suppl. Fig. 24. **Clock times of stool samples and sleep and feeding history at each stool sample time**. Visualization is limited to samples with directly preceding feeding and sleeping information.

Suppl. Fig. 25. **Alpha diversity and age**. Alpha diversity metrics measured by observed features, Shannon entropy, Pielou evenness and Faith phylogenetic diversity. **a** Regression lines (95% confidence intervals) of alpha diversity along age in the samples (all p<0.001). **b** Coefficient plots showing sleep and feeding history effects on alpha diversity. Significant associations between all alpha diversity metrics and age (p<0.001). Limited evidence of an association between Faith phylogenetic diversity (features biodiversity) and sex (p<0.1). Dots are the coefficients and error bars represent the 95% confidence intervals.

Suppl. Fig. 26. **Alpha rarefaction curves and sample retention**. Rarefaction curves represent the mean Shannon diversity per sample (calculated across 10 iterations) over 20 sequencing depth intervals. The primary y-axis (left) denotes the Shannon diversity index, while the secondary y-axis (right) indicates the number of samples retained at each depth (total n = 187). The horizontal dashed line marks a retention threshold of 163 samples, and the vertical black line indicates the chosen rarefaction depth of 3,035 reads.

Suppl. Table 1. **Results of random forest classifiers**. Classifiers predicting the babySQUID binarized at the median (0 if below median, 1 if > median). Using the relative abundances of ASVs to predict the babySQUID, the model achieved an average accuracy of 58.6% (±24.9%) and a weighted F1 score of 0.551 (±0.277). Predictive performance improved further after k-merizing the feature table (k-mer length = 16) and selecting the top 20 features. This approach increased the accuracy to 63.3% (±8.46%) and the weighted F1 score to 0.609 (±0.103), indicating both enhanced performance and reduced variability (same results obtained when top features selected based on their frequency and TF-IDF score). Cross-validation methods: (1) GroupShuffleSplit (n=5): generates a user-determined number of random test splits, each with a user-determined fraction of unique groups; (2) GroupKFold (n=5): K-fold iterator variant with non-overlapping groups; (3) LeaveOneGroupOut (n=18 infants): leave-one-out approach (i.e., for each of N folds, train on N-1 infants and test on the Nth, where N = number of infants). Accuracy = (number of correct predictions) / (total number of predictions). F1 Score = harmonic mean of precision and recall (precision = proportion of true positives in all predicted positives; recall = proportion of (correctly) predicted positives out of all true positives).

| **Feature Table** | **Cross-Validation** | **Mean accuracy (± SD)** | **Mean weighted F1 Score (± SD)** |
| --- | --- | --- | --- |
| **ASVs (426 features)** |  |  |  |
|  | GroupShuffleSplit | 0.468 ± 0.174 | 0.381 ± 0.225 |
|  | GroupKFold | **0.586 ± 0.249** | **0.551 ± 0.277** |
|  | LeaveOneGroupOut | 0.593 ± 0.402 | 0.574 ± 0.424 |
| **K-mers (k=10)** |  |  |  |
|  | GroupShuffleSplit | 0.408 ± 0.156 | 0.372 ± 0.159 |
|  | GroupKFold | 0.424 ± 0.105 | 0.370 ± 0.100 |
|  | LeaveOneGroupOut | 0.306 ± 0.417 | 0.298 ± 0.419 |
| **K-mers (k=20)** |  |  |  |
|  | GroupShuffleSplit | 0.598 ± 0.042 | 0.595 ± 0.040 |
|  | GroupKFold | **0.633 ± 0.085** | **0.609 ± 0.103** |
|  | LeaveOneGroupOut | 0.574 ± 0.409 | 0.576 ± 0.416 |
| **K-mers (k=50)** |  |  |  |
|  | GroupShuffleSplit | 0.438 ± 0.061 | 0.431 ± 0.082 |
|  | GroupKFold | 0.548 ± 0.080 | 0.493 ± 0.137 |
|  | LeaveOneGroupOut | 0.491 ± 0.362 | 0.465 ± 0.375 |
| **TF-IDF k-mers (k=10)** |  |  |  |
|  | GroupShuffleSplit | 0.416 ± 0.161 | 0.389 ± 0.180 |
|  | GroupKFold | 0.510 ± 0.129 | 0.455 ± 0.158 |
|  | LeaveOneGroupOut | 0.444 ± 0.412 | 0.419 ± 0.421 |
| **TF-IDF k-mers (k=20)** |  |  |  |
|  | GroupShuffleSplit | 0.544 ± 0.079 | 0.536 ± 0.092 |
|  | GroupKFold | **0.633 ± 0.085** | **0.609 ± 0.103** |
|  | LeaveOneGroupOut | 0.574 ± 0.374 | 0.557 ± 0.389 |
| **TF-IDF k-mers (k=50)** |  |  |  |
|  | GroupShuffleSplit | 0.517 ± 0.103 | 0.520 ± 0.102 |
|  | GroupKFold | 0.548 ± 0.080 | 0.505 ± 0.141 |
|  | LeaveOneGroupOut | 0.546 ± 0.359 | 0.539 ± 0.375 |
